# A Highly Stable Plastidic-Type Ferredoxin-NADP(H) Reductase in the Pathogenic Bacterium *Leptospira interrogans*

**DOI:** 10.1371/journal.pone.0026736

**Published:** 2011-10-24

**Authors:** Daniela L. Catalano-Dupuy, Matías A. Musumeci, Arleth López-Rivero, Eduardo A. Ceccarelli

**Affiliations:** Molecular Biology Division, Instituto de Biología Molecular y Celular de Rosario (IBR), CONICET, Facultad de Ciencias Bioquímicas y Farmacéuticas, Universidad Nacional de Rosario, Rosario, Argentina; University of Delhi, India

## Abstract

*Leptospira interrogans* is a bacterium that is capable of infecting animals and humans, and its infection causes leptospirosis with a range of symptoms from flu-like to severe illness and death. Despite being a bacteria, *Leptospira interrogans* contains a plastidic class ferredoxin-NADP(H) reductase (FNR) with high catalytic efficiency, at difference from the bacterial class FNRs. These flavoenzymes catalyze the electron transfer between NADP(H) and ferredoxins or flavodoxins. The inclusion of a plastidic FNR in *Leptospira* metabolism and in its parasitic life cycle is not currently understood. Bioinformatic analyses of the available genomic and proteins sequences showed that the presence of this enzyme in nonphotosynthetic bacteria is restricted to the *Leptospira* genus and that a [4Fe-4S] ferredoxin (LB107) encoded by the *Leptospira* genome may be the natural substrate of the enzyme. *Leptospira* FNR (LepFNR) displayed high diaphorase activity using artificial acceptors and functioned as a ferric reductase. LepFNR displayed cytochrome *c* reductase activity with the *Leptospira* LB107 ferredoxin with an optimum at pH 6.5. Structural stability analysis demonstrates that LepFNR is one of the most stable FNRs analyzed to date. The persistence of a native folded LepFNR structure was detected in up to 6 M urea, a condition in which the enzyme retains 38% activity. *In silico* analysis indicates that the high LepFNR stability might be due to robust interactions between the FAD and the NADP^+^ domains of the protein. The limited bacterial distribution of plastidic class FNRs and the biochemical and structural properties of LepFNR emphasize the uniqueness of this enzyme in the *Leptospira* metabolism. Our studies show that in *L. interrogans* a plastidic-type FNR exchanges electrons with a bacterial-type ferredoxin, process which has not been previously observed in nature.

## Introduction

Ferredoxin-NADP(H) reductases (FNRs) are ubiquitous monomeric enzymes that contain a noncovalently bound FAD as a prosthetic group. To date, the participation of FNRs has been documented in processes as dissimilar as photosynthesis, steroid hydroxylation, nitrate reduction, anaerobic pyruvate assimilation and fatty acid desaturation. The involvement of FNR has been also documented in xenobiotic detoxification, amino acid and deoxyribonucleotide synthesis, iron-sulfur cluster biogenesis and in the regulation of several metabolic pathways [1]–[4].

The biological catalytic FNR activity comprises the reversible electron transfer between NADP(H) and different low potential one-electron donors (e.g., ferredoxin, flavodoxin, adrenodoxin, heme-oxygenase and iron) [1]–[7].

The aforementioned reductase activities are performed, in nature, by enzymes that belong to two unrelated protein families [2], [3]. One of these families includes adrenodoxin reductases, some bacterial homologues and bacterial oxygenase-coupled NADH-ferredoxin reductases. Enzymes of the above group have been found mainly in the mitochondria of eukaryotic organisms and in some bacteria such as *Mycobacterium tuberculosis* and *Pseudomonas*. The other group, known as the plant-type FNR family, contains FNRs that are easily identified by the presence of highly conserved amino acid clusters located in the FAD and NADP(H) binding domains [2]. This group is subdivided into bacterial and plastidic FNR classes. Whereas plastidic FNRs display turnover numbers that are related to the photosynthesis needs (200–600 s^−1^), bacterial FNRs are much less active, with turnover numbers that are 20- to 100-fold lower than those of their plastidic counterparts [1], [2], [8].

The low sequence homology between both subgroups (plastidic and bacterial FNRs) weakens the possibility of performing phylogenetic analyses of all group members; however, a common ancestor for all plant-type FNRs can be expected. We have previously assigned the FNR from *Leptospira interrogans* (LepFNR) to the plastidic FNR class [2], and this was confirmed by resolution of the LepFNR crystal structure [9]. The presence of a plastidic FNR in *Leptospira* might be explained as the result of lateral gene transfer, and this was probably selected to provide the bacterium with some adaptive advantages. Plastidic-type FNRs have been also found in *Plasmodium falciparum* [10], [11] and *Toxoplasma gondii* [12], [13].

*L. interrogans* is a parasitic bacterium that infects humans and causes leptospirosis, which is also known as Weil's disease. Leptospiral infection in humans results in a range of symptoms. The infection is often misdiagnosed due to the wide range of symptoms, which include high fever, severe headache, chills, muscle aches, jaundice, and others. Complications may include several organ malfunction, meningitis, extreme fatigue, respiratory distress, liver and renal pathologies, which may often produce death. *Leptospira* infects other mammals as well, including rats, cattle, horses, pigs and dogs, which are natural reservoirs. This zoonotic disease is an important, emerging worldwide-distributed infection that is responsible for numerous human deaths. The disease is spreading from its traditional rural areas to poor urban slum communities and neighborhoods with deteriorated environmental conditions [14].

The inclusion of a highly efficient FNR in *Leptopspira* metabolism may be an important element of its parasitic life. The question of how this enzyme participates in the *Leptospira* metabolism is then logical. Several roles have been proposed in which these plastidic enzymes may fulfill some specific metabolic needs in parasitic organisms such as *T. gondii* and *P. falciparum* [3], [15], [16].

Despite the great divergence that the *Leptospira* and apicomplexan FNRs display at the primary structural level, with respect to those found in plants, they have conserved high catalytic rates [3], [9]. Therefore, the efficiency of these enzymes may have been maintained through selective pressure and may play an essential role in the life of the parasites. Based on these arguments, it has been proposed that FNR is a relevant target for specific drug development against parasitic apicomplexan [3], [15], [16].

Recently, much effort has been placed into the study of the structure and function of the plastidic FNRs from apicomplexan organisms [3]. Along these lines, the *L. interrogans* FNR has been cloned and its crystal structure has been resolved [9].

With the aim of determining whether *Leptospira*, besides photosynthetic bacteria and apicoplexan parasites, are the unique bacteria that contain a highly efficient plastidic FNR, we performed a screen for this enzyme in all bacterial genomes sequenced to date. Representative members of each taxon have been selected and used to perform a phylogenetic analysis to investigate how each is related to the plant and *Leptospira* FNRs. To infer the metabolic importance of the *L. interrogans* FNR, we investigated its functional and structural properties. We found that this plastidic enzyme displays particular features that are unique and not found in any of the other FNRs studied to date.

## Results and Discussion

### Sequence alignment and phylogeny of plastidic FNRs

**Figure 1:**
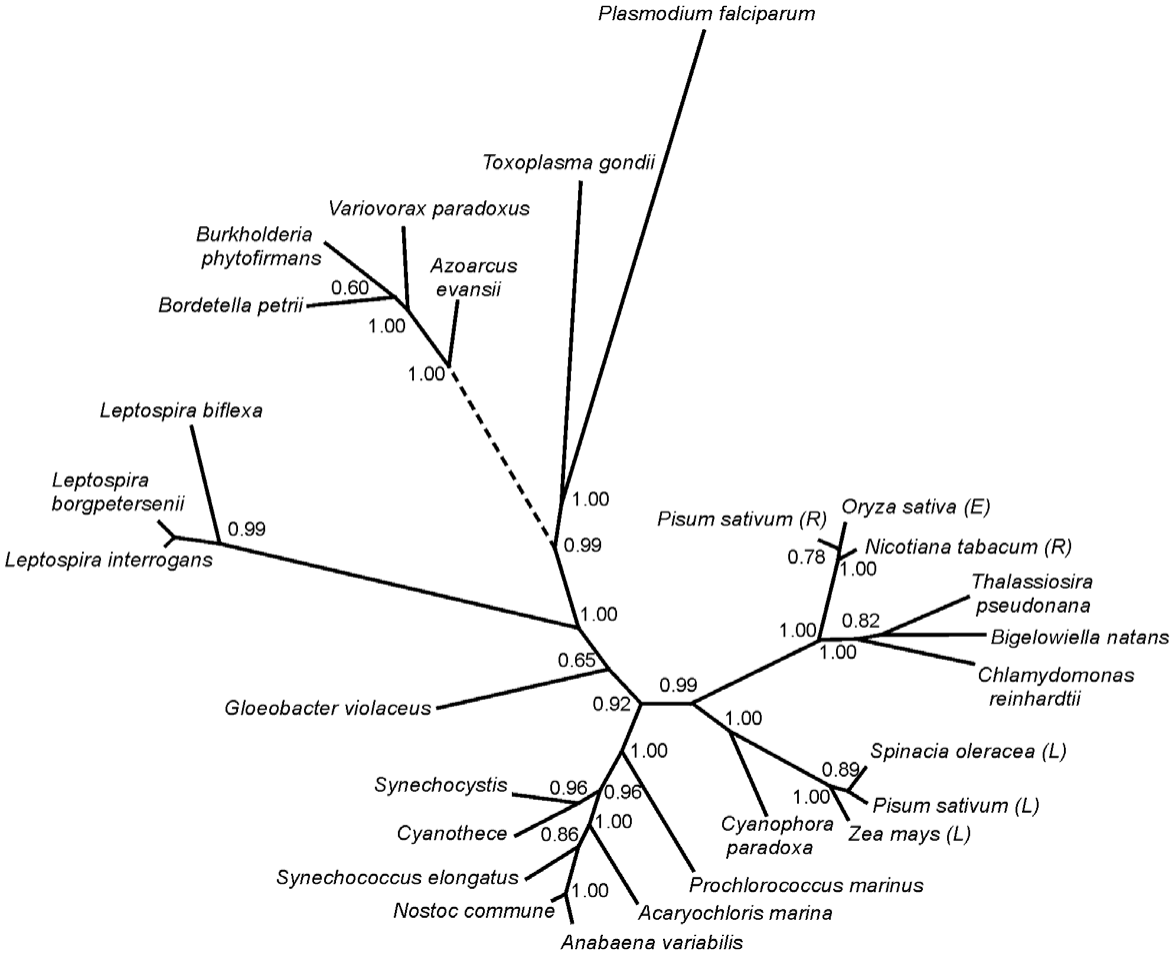
Phylogenetic relationship between plastidic-type FNRs found in bacteria and photosynthetic organisms. This tree was constructed as described in the Materials and Methods section. The distance between two sequences can be obtained by adding the lengths of the connecting branches. The letters in brackets indicate reductases from leaf (L), embryo (E) or root (R). The dashed line indicates an inference made in this work. The database accession numbers of different sequences under study are indicated in Figure 1S legend.

The complete DNA database that is available at the NCBI was searched using the tBLASTn method with the FNR amino acid sequences from *L. interrogans*, the mature region of the *Pisum sativum* (pea) from leaf, *Escherichia coli* and *Rhodobacter capsulatus* as query sequences. We also analyzed all genomic sequences that have been previously identified as putative FNRs. We observed that none of the bacterial genomes contain sequences with an acceptable degree of similarity to plastidic FNRs, with the exception of members of the *Cyanobacteria* phylum and the *Leptospiraceae* family. No plastidic FNRs were found in the genomes of the closely related members of the *Brachyspiraceae* and *Spirochaetaceae* (i.e., *Treponema, Borrelia*) families. Some members of the *Leptospiraceae* family, such as *Leptospira biflexa*, contain both a plastidic and a bacterial FNR. In contrast, we were unable to identify a bacterial-type FNR in the *Leptospira* pathogenic species, and only the plastidic-type was found. In our analysis, we also identified a component of the aromatic degradation pathway that is found in the Betaproteobacterias of the *Rhodocyclaceae* family (i.e., BoxAB) of *Azoarcus evansii* [17] and in members of the order *Burkholderiales* (i.e., *Bordetella*, *Burkholderia*, which was previously a part of *Pseudomonas*, *Ralstonia*, *Variovorax*, *etc.*) (Fig. 1). These organisms share the capability to efficiently degrade organic compounds, and some of these are important pathogens. BoxAB has been implicated in the aerobic benzoyl-CoA pathway in *Azoarcus evanssi*. The BoxAB purified enzyme catalyzes the O_2_-dependent oxidation of NADPH with a strong preference against NADH [18], showing a substrate specificity characteristic of ferredoxin-NADP^+^ reductases. Recently, it has been proposed that the carboxyl terminal domain of this enzyme should be named benzoyl-CoA NADPH-oxygen oxidoreductase [17]. This protein contains an amino terminal domain that is related to an Fe-S binding protein and a carboxy terminal region of approximately 300 amino acids. We propose that the carboxyl terminal domain of this enzyme should be classified as a plant-type reductase. By sequence homology (Supporting Fig. S1), we found that the six conserved peptide segments that define the plant-type FNR structural family are properly aligned. A distinguishing feature among plastidic FNRs is the common motif SLCV(K/R)(R/Q)(L/A) [9]. In LepFNR, this region contains the basic amino acids Lys and Arg, which are close to the FAD binding site. The BoxAB type enzymes are more closely related to plastidic enzymes at this region, and they contain the sequence SLTV, which is followed by two positively charged amino acids. The aromatic residue that interacts with the adenosine moiety in plastidic-type FNRs was not found in BoxAB enzymes. However, using the SWISS-MODEL protein structure homology-modeling server, it was possible to obtain a good quality estimation of the structure that resembles those of the plastidic class of FNRs (Supporting Fig. S2) in which the loop that extends the FAD conformation is present. Thus, this might be a new type of plastidic reductase.

We subsequently analyzed the phylogenetic relationships of representative members of the plastidic FNR class. Figure 1 shows a consensus tree of a matrix of 27 selected taxa with Bayesian posterior probabilities of 60% or more (Fig. 1 and Supporting Fig. S1). The plastidic *Leptospira* enzyme shares a common ancestor with reductases found in the plants and *Cyanobacteria*, and they probably diversified very early in the evolution. Most of the plastidic class enzymes are found in eukaryotes belonging to the *Viridiplantae* kingdom. Other members were also found in the *Cyanobacteria* phylum.

The plastidic-type FNRs found in bacteria in the genus *Leptospira*, of which some are entirely parasites of vertebrates, segregated with FNRs found in the apicomplexan eukaryotic parasites including *T. gondii* and *P. falciparum*, which display highest sequence similarity with the root-type FNRs [19]. LepFNR is neither clearly related to the root nor the leaf enzymes and seems to display some structural features from both groups. Two basic residues at positions 82 and 85, distinctive of leaf FNRs (numbers as in mature pea leaf FNR) are serine/asparagine in the primary structure of root FNRs (Supporting Fig. S3). K82 is conserved in LepFNR (K84) while the second positively charge residue is replaced by a threonine (T92), as in FNRs from plant embryos. These residues have been implicated in the interaction of the root and leaf enzymes with their protein substrates [20]. In *T. gondii* and *P. falciparum* these residues are replaced by a leucine and a cysteine next to a large sequence insertion. It has been proposed that the apicoplast evolved via an endosymbiotic event, providing a plastidic-type FNR during this process among other proteins [21]. At variance, in *Leptospira*, the plastidic FNR probably arrived from a lateral gene transfer event. Thus, segregation of the orthologous apicomplexan FNRs and the paralogous members from *Leptospira* may be an indication of some common properties of these enzymes and not an evolutionary relationship. We have strengthened our phylogenetic analysis using the phylogenetic packed Phylip 3.66, applying Neiborgh-Joining and bootstrapping. A similar tree was obtained (Supporting Fig. S4). In both trees the BoxAB type enzymes clustered near the *Leptospira* group. These results are not completely unexpected because it has been suggested that the major evolutionary changes within prokaryotes have occurred in a directional manner, *Spirochetes* being the older species than *Betaproteobacteria* [22].

### Functional characterization of LepFNR

LepFNR showed high diaphorase activity using bi-electronic artificial acceptors such as potassium ferricyanide with a *K*_m_ and *k*_cat_ for NADPH of 14.5 µM and 258.2 s^−1^, respectively (Table 1). These parameters were comparable with values obtained for the pea leaf FNR in a parallel experiment, in which the *K*_m_ and *k*_cat_ values were 15.0 µM and 324.8 s^−1^, respectively, indicating that despite the divergence in primary structure, LepFNR displays a high catalytic competence. Values for *k*_cat_ and *K*_m_ for NADPH of 700 e^−^ eq/s (equivalent to 350 s^−1^) and 34 µM, respectively, were determined for the *T. gondii* FNR [23]. The *P. falciparum* enzyme displayed a lower catalytic efficiency as observed in bacterial-type FNRs; values for *k*_cat_ and *K*_m_ for NADPH of 250 e^−^ eq/s (equivalent to 125 s^−1^) and 36 µM, respectively, were reported [10]. Diaphorase activity of root and leaf FNRs from maize was measured using DCPIP as electron acceptor. Root FNR showed a *k*_cat_/*K*_m_ value for NADPH higher than leaf isozyme and this was attributed mainly to a large difference in their *K*_m_ values (0.39 and 4.4 µM, respectively) [20]. When diaphorase activity was assayed using LepFNR and NADH as electron donor instead of NADPH, no electron transfer was detected with potassium ferricyanide. These results indicate that, similar to the other reductases [1], LepFNR has a strong preference for NADP(H) and is a poor NAD(H) oxidoreductase.

**Table 1:** Kinetic parameters of the different studied FNRs^a^.

Enzyme		NADPH-K_3_Fe(CN)_6_ diaphorase kinetic parameters			Ferric reductase activity		Oxidase activity	
	*K*_d_^NADP+^	*K*_m_	*k*_cat_	*k*_cat_/*K*_m_	Aerobic	Anaerobic	Aerobic	Anaerobic
	(µM)	(µM)	(s^−1^)	(µM^−1^ s^−1^)	(s^−1^)	(s^−1^)	(s^−1^)	(s^−1^)
LepFNR	13.2±3.1	14.5±1.7	258.2±13.7	17.8	0.83±0.07	1.75±0.05	0.66±0.02	≤0.02
Pea leaf FNR	11.3±2.1	15.0±1.6	324.8±16.5	21.7	0.61±0.07	0.75±0.03	1.75±0.07	≤0.01
*E. coli* FNR	7.6±2.2	8.3±0.5	38.2±2.3	5.0	0.67±0.04	0.64±0.03	0.50±0.08	≤0.02

a Parameters were calculated as described in the Materials and Methods section.

It has been previously shown that the *Pseudomonas* [6] and *E. coli* FNRs function as flavin and ferric reductases [7]. We investigated whether LepFNR uses ferric-EDTA or ferric-citrate as terminal electron acceptors. The FNRs from *Leptospira*, pea and *E. coli* all display ferric reductase activity at similarly low levels. The LepFNR has double the ferric reductase activity in anaerobic conditions as compared with the other tested reductases. LepFNR displayed an oxidase activity similar to that observed with the bacterial enzyme and 38% of the activity measured for the plant leaf enzyme (Table 1). Ferric reductase activity is probably not the main function of LepFNR in *Leptospira*; however, the significance of this activity in *Leptospira* metabolism should not be underestimated. Recently, a model of cytosolic iron release in bacteria was proposed in which an unspecific reduction by the ferredoxin-NADP^+^ reductase may occur [24]. It has been observed that *L. interrogans* requires a functional heme oxygenase to grow in the presence of hemoglobin [25]. Interestingly, the same authors determined that *Leptospira* prefers to use ferrous iron when multiple sources of iron are present [26]. Therefore, we suggest that LepFNR may be involved in the reduction from ferric to ferrous iron turning it in an utilizable form, although this may not be the main metabolic pathway involved in this process.

**Figure 2:**
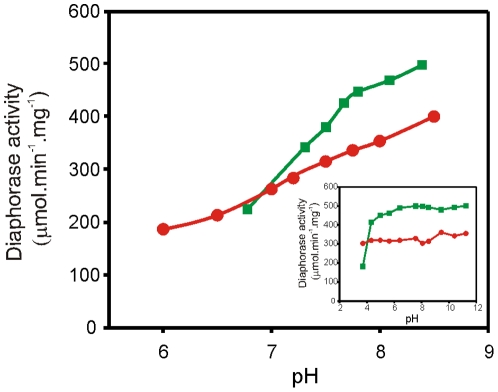
Enzyme activity as a function of pH. NADPH-ferricyanide diaphorase activity of FNRs was assayed at different pH values. Inset: NADPH-ferricyanide diaphorase activity assayed at pH 8.0 after incubating the FNR samples at the indicated pH values for 30 min at 30°C. LepFNR (red circles); pea leaf FNR (green squares).

The dependency of the LepFNR diaphorase activity on pH is shown in Figure 2. The increase of ferricyanide diaphorase activity with pH was higher for pea leaf FNR than LepFNR. At pH 6.75, both enzymes displayed the same activity, while at pH 8, a maximal proportional difference was observed, with the pea FNR activity being 35% higher than that of LepFNR. To assess if these differences in activity were due to differences in protein stability, the FNR activity as a function of pH was further investigated. Pea leaf FNR and LepFNR were incubated at different pHs for 30 minutes at 30°C, and then diaphorase activity measurements were performed at pH 8. At pH 3.7, the pea leaf FNR activity dropped approximately 60%, and we observed no inactivation of LepFNR (Fig. 2, inset). Incubation at 37°C for 2 hours at the same pH provokes a 50% decrease in the LepFNR catalytic activity, whereas this treatment almost inactivated pea leaf FNR (not shown). These results suggest that LepFNR has increased stability at acidic pH as compared to the pea leaf enzyme, while both proteins are equally stable over a wide pH range of 5–11.

**Figure 3:**
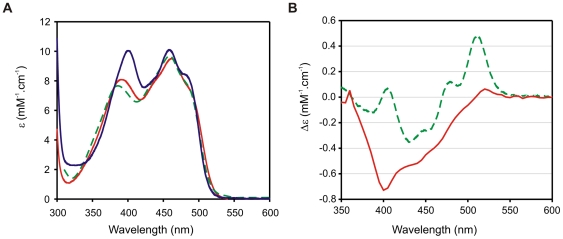
Spectral properties. (A) UV-visible spectra of FNRs. (B) Difference absorption spectrum elicited by NADP^+^ at saturating concentration (400 µM). LepFNR (red solid line), pea leaf FNR (green dashed line) and *E. coli* FNR (blue solid line).

Flavoproteins are usually obtained in its oxidized state and therefore show a characteristic FAD absorption spectrum. Figure 3A shows the spectra of the pea leaf FNR, *E. coli* FNR and LepFNR, all essentially in oxidized states. The LepFNR spectrum is similar to that of pea leaf FNR, with a slight difference in the intensity of the minor peaks, corresponding to 385 nm for the plant leaf enzyme and 390 nm for the LepFNR. From the spectral data, we obtained a A_275_/A_459_ ratio of eight for the LepFNR and we estimated an extinction coefficient of 9.5 mM^−1^ cm^−1^ at 459 nm.

We determined the dissociation constant for the LepFNR.NADP^+^ complex using differential absorption spectroscopy. The final spectrum obtained at saturating concentration of NADP^+^ is shown in Figure 3B. For comparison, a differential spectrum obtained with pea leaf FNR and a saturating concentration of NADP^+^ is also shown. We estimated a *K*_d_ value of 13.2 µM for the LepFNR·NADP^+^ complex (Table 1).

It has been observed that FNRs are promiscuous enzymes with respect to their protein substrates ferredoxins or flavodoxins. To assess whether LepFNR interacts with protein substrates from other sources, cytochrome *c* reductase activity was measured using *P. sativum* leaf ferredoxin. The observed activity levels were 30-fold lower than those observed with pea leaf FNR (2.5 versus 75 s^−1^). Additionally, no kinetic parameters were determined because the reaction showed a linear correlation with the concentration of pea leaf ferredoxin for up to 70 µM, indicating that the affinity of the LepFNR and pea leaf ferredoxin is considerably low. Using ferredoxin and flavodoxin A from *E. coli*, for up to a concentration of 30 µM each, no cytochrome *c* activity was detected.

When the LepFNR crystal structure was fit with the maize leaf FNR structure in complex with ferredoxin (PDB 1gaq), we observed that LepFNR amino acids P79, E80 and K81 may interfere with the pea ferredoxin binding (Supporting Fig. S5). These amino acids belong to a particular big loop that ranges from I66 to S100 in LepFNR. Multialigned sequences (see Supporting Fig. S1) show that this region of LepFNR contains at least six inserted amino acids that are not found in the other plastidic FNRs, with the exception of *Plasmodium* and *Toxoplasma* FNRs, which have 30 and 29 inserted amino acids, respectively. It has been shown that deletion of this species-specific subdomain in the *Toxoplasma* FNR resulted in an enzyme form that displayed a 10-fold decreased affinity for Fd [27]. By site-directed mutagenesis, it has been demonstrated that a lysine residue (K75 in *Anabaena viariabilis* FNR) is essential for productive binding of the ferredoxin substrate [28]. Substitution of this positively charged residue by neutral amino acids increased the *K*_d_ value for ferredoxin 50 to 100 times. Moreover, replacement of this residue by a negatively charged amino acid results in substrate binding capability loss [28]. This lysine residue is conserved in all species with the exception of *T. gondii*, *P. falciparum*, *Chlamydomonas reinhardtii* and non-photosynthetic isoforms from plants [19]. This position corresponds to a threonine residue (T92) in LepFNR [29]. The other positively charge residue (K82 in pea leaf FNR) which has been implicated in the interaction of the leaf enzymes with their protein substrate ferredoxin [20] is conserved in LepFNR as mentioned before.

It is clear that the substrate or substrates for the plastidic-type LepFNR should be considerably different from those already identified for plastidic FNRs. Two genes, LA4086 and LB107, were identified as putative ferredoxins from the completely sequenced *L. interrogans* genome. The sequence alignment analyses of these ferredoxins showed that LA4086 corresponds to a [2Fe-2S] ferredoxin with a thioredoxin-like fold [29], whereas LB107 contains typical ferredoxin cysteine residues with [4Fe-4S] centers [29] (Supporting Fig. S6A and B). Neither of these proteins are plant-type ferredoxins, the common partners of plastidic class FNRs. Ferredoxins homologous to L4086 have been previously characterized [30], [31]. Evidence has suggested that some of these ferredoxins may be involved in nitrogen fixation metabolism [31], which may not be the case in *Leptospira*.

Alternatively, when the ferredoxin LB107 was aligned with the iron-sulfur component of the *Azoarcus evansii* BoxAB enzyme, we observed only a relative overall homology. However, all of the relevant residues that are involved in cluster formation are conserved among both proteins (Supporting Fig. S6C). Moreover, we were unable to find a flavodoxin in the *L. interrogans* genome.

We therefore decided to clone and express the genes coding for LA4086 and LB107 ferredoxins. Both proteins were purified with its Fe-S cluster properly bound by co-expressing the biogenesis iron-sulfur system (ISC) from *E. coli* [32]. LB107 ferredoxin required an O_2_-free atmosphere during the purification protocol.

**Figure 4:**
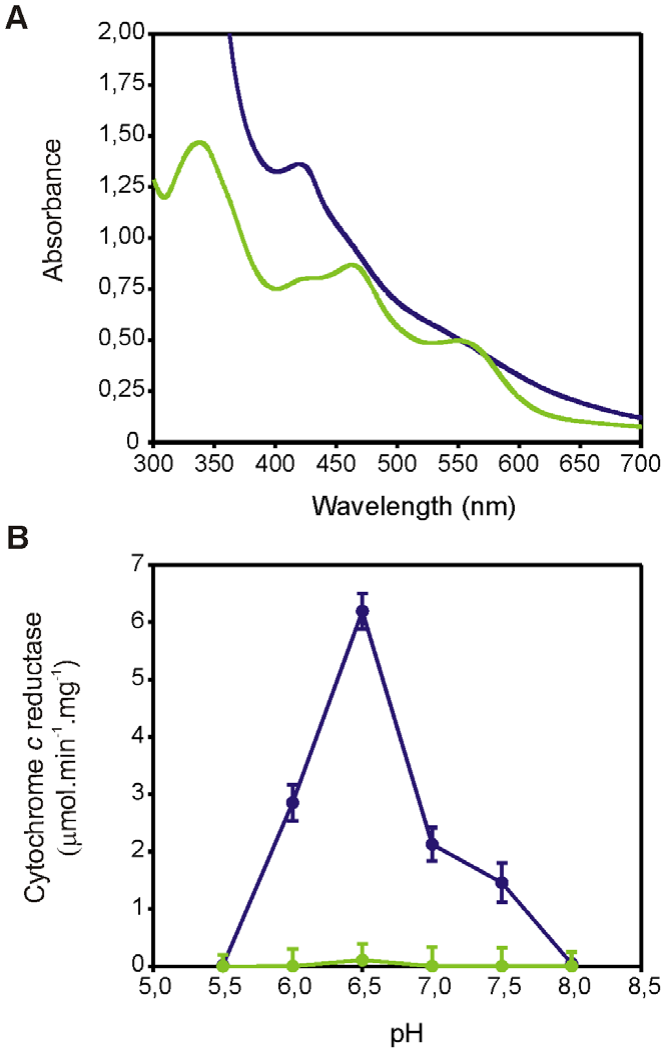
Ferredoxins from *L. interrogans*. (A) UV-visible spectra of [2Fe-2S] thioredoxin-like ferredoxin (LA4086, green) and [4Fe-4S] ferredoxin (LB107, blue) from *L. interrogans*. (B) Cytochrome *c* reductase activity assayed with LepFNR and LA4086 (green) or LB107 (blue) ferredoxins at different pH values.

The electronic absorption spectra of oxidized LA4086 and LB107 ferredoxins are shown in Figure 4A. LA4086 spectrum exhibits absorption maxima at 339, 422, 465, and 556 characteristic of ferredoxins containing a [2Fe-2S] cluster [31]. At difference, the LB107 ferredoxin displayed a broad spectrum with a band at 425 nm, which is typical of [4Fe-4S] and [3Fe-4S] ferredoxins [5], [33]. The NADPH-cytochrome *c* reductase activity of LepFNR with both ferredoxins was measured under anaerobic conditions (Figure 4B). Negligible activity was obtained with LepFNR and the LA4086 ferredoxin. On the contrary, a maximal specific activity of 6.13 µmol of cytochrome *c* reduced/mg of LepFNR/min was obtained at pH 6.5 with LB107 ferredoxin. This value is more than 7 times higher than the corresponding activity observed with the seven-iron ferredoxin I and the FPR from *Azotobacter vinelandii* [34]. As shown in Figure 4B, the electron transfer is pH-dependent with lower values at higher pHs. Similar results were obtained for the related *A. vinelandii* ferredoxin probably due to the change of the ferredoxin reduction potential to a close value to that of the ferredoxin-NADP^+^ reductase [34].

Complex formation between seven-iron ferredoxin and bacterial FNRs has been previously observed [35], though not with plastidic-type FNRs. Our studies show that in *L. interrogans* a plastidic-type FNR exchanges electrons with a bacterial-type [4Fe-4S] ferredoxin, process which has not been previously observed in nature. These results do not preclude the possibility that LepFNR exchange electrons with other protein substrates as usually occurs in other organisms.

### Overall structure and stability properties of the enzyme

**Figure 5:**
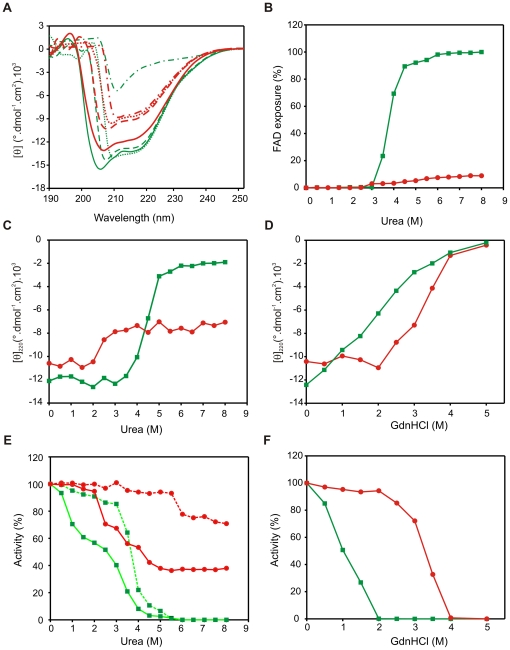
Effect of urea and guanidine hydrochloride on structural integrity of FNRs. (A) Circular dichroism spectra of LepFNR (red lines) and pea leaf FNR (green lines) registered in the absence (solid lines) and presence of 1 M (dashed lines), 3 M (dotted lines) and 6 M (dotted and dashed lines) urea. (B) FAD exposure determined by the increase in protein fluorescence with urea addition. One-hundred percent FAD exposure was considered as the fluorescence displayed by completely denaturated enzyme with 2% SDS. (C) (D) Ellipticity at 220 nm plotted as a function of urea and GdnHCl concentration, respectively. (E) NADPH-ferricyanide diaphorase activity assayed in the absence and presence of urea. In all cases, the enzyme samples were incubated at the indicated urea concentrations for 1 h at 25°C. Then the activity was measured in medium containing an urea concentration that was equivalent to that in the incubation medium (solid lines) or by diluting the sample in a reaction medium without the chaotrope (dashed lines). (F) NADPH-ferricyanide diaphorase activity elicited by the FNRs incubated for 1 h at 25°C in GdnHCl and diluted in a reaction medium without the chaotrope. LepFNR (red circles); pea leaf FNR (green squares).

The circular dichroism (CD) spectra in the far-UV region were registered for LepFNR and the pea leaf enzyme at different urea concentrations (Figure 5A). Figure 5C shows the change in ellipticity at 220 nm as a function of urea concentration for LepFNR and pea leaf FNR. FAD and NADP^+^ binding domains both contain protein helical structures. Consequently, signals may arrive from changes in any domain. The unfolding of pea FNR shows a typical cooperative process with an apparently complete unfolding of the molecule. On the contrary, urea-induced unfolding of LepFNR shows no changes at a low urea concentration (0–2 M urea) and the persistence of structures up to 8 M urea. This observation may be an indication that LepFNR (or part of its structure) is considerably more stable than the pea leaf FNR. It has been previously observed that the FNRs from *T. gondii* [36], *Mycobacterium* [37], pea leaf [38] and maize leaf [39] can be completely unfolded by 6 M urea treatment.

FAD fluorescence is strongly quenched in FNRs due to interaction of the flavin with the protein scaffold [38]. As shown in Figure 5B, only a slight increase in FAD exposure was observed for LepFNR as a function of urea concentration as evidence that the prosthetic group remains bound to the enzyme and no considerable disruption of the tridimensional structure of the FAD binding domain has occurred. In contrast, the pea leaf FNR FAD fluorescence rose after 3 M urea which indicates the release of the flavin. The shape of the curve is coincident with that obtained by the urea unfolding followed by CD-spectroscopy. Therefore, it may be concluded that in the case of pea leaf FNR cooperative unfolding involves both the FAD and the NADP(H) binding domains, which does not seem to be the case for LepFNR. Likewise, the global unfolding induced by urea in the maize leaf FNR is a cooperative process in which unfolding of the secondary structure and the release of FAD occur concomitantly [39].

The diaphorase activities of the pea leaf and *Leptospira* FNRs were also studied as a function of urea concentration using two different conditions: after 1 hour of enzyme incubation with several urea concentrations, i) the activity was measured in a medium containing an urea concentration equivalent to that in the incubation medium (solid lines in Fig. 5E), or ii) the activity was determined in the absence of urea (dashed lines in Fig. 5E). Of note, LepFNR (red circles) retains 38% activity in the presence of 8 M urea, while pea leaf FNR is completely inactive in urea concentrations higher than 6 M (green squares). Moreover, LepFNR recovered 70% of its activity after incubation with 8 M urea when the reaction medium did not contain this chaotrope. Thus, the prosthetic group stood firmly bound to the protein during the urea treatment, although the enzyme decreased its catalytic activity. To retain FAD binding capacity, the enzyme inter-domain region, where the isoalloxazine moiety is located, should remain folded. During the unfolding of multidomain proteins, one domain can remain stable while other domain completely unfolds, or contains folded and unfolded subdomains [40], which may be the case for LepFNR in high urea concentrations.

Measuring hydrogen/deuterion exchange by heteronuclear NMR analysis of the maize leaf FNR, it has been observed that the α-helix of the NADP^+^ binding domain, located at the interface between the FAD and the NADP^+^ domains, is one of the most protected and chaotrope resistant regions in the protein [39]. The same authors determined that the NADP^+^ binding domain of maize leaf FNR is preferentially destabilized in urea, probably by affecting the flexible subdomain. This flexible region has been observed in several FNRs [41]–[43]. Accordingly, the LepFNR crystal structure reveals that parts of the NADP^+^ binding domain is highly mobile, as indicated by the obtained B factors [9]. This mobility may be important for obtaining high catalytic efficiency in FNRs [42], [44].

Figure 5D shows the unfolding curves of pea leaf FNR and LepFNR that were obtained by monitoring the CD signal at 220 nm as a function of increasing guanidinium chloride concentration. Unlike the LepFNR urea unfolding, no evidence of remaining folded states was observed at high chaotrope concentrations. Pea leaf FNR shows a continuous decrease in secondary structure concomitant to the increase in chaotrope concentration. At 2 M guanidinium chloride, the pea leaf FNR was completely inactive, with 50% loss in CD signal (Fig. 5D and F). In contrast, LepFNR shows a sigmoidal transition, in which the loss of the CD signal is well correlated with the activity disappearance. In this case, denaturation of the LepFNR NADP^+^ binding domain may trigger the collapse of the FAD binding domain, which is similar to what was previously observed in the *T. gondii* FNR [36].

The thermal stability of LepFNR and pea leaf FNR at pH 7.5 were studied and the thermodynamic parameters were calculated (Table 2). The temperature of maximal stability (T_x_) and the internal free energy at T_x_ for LepFNR are 5 degrees centigrade and 3.13 kcal/mol higher, respectively, than the values obtained for pea leaf FNR. It has been observed that thermal unfolding of maize root and spinach leaf FNR is irreversible and that leaf enzyme is more stable than the root isoform [45].

**Table 2:** Thermal unfolding parameters of the different studied FNRs^a^.

Enzyme	T_m_ b	T_x_ c	Δ*G*_(Tx)_ d
	(°C)	(°C)	(kcal/mol)
LepFNR	68.1±0.9	43.8±1.2	7.75±0.9
Pea leaf FNR	61.3±1.3	38.8±0.7	4.62±0.8
*E. coli* FNR	56.8±0.8	36.5±1.1	2.23±0.5

a Parameters were calculated as described in the Materials and Methods section.

b T_m_: melting temperatures for the folding-unfolding transitions.

c T_x_: temperature of maximal stability that corresponds to the temperature in which the internal free energy is maximal.

d Δ*G*_(Tx)_: value of the internal free energy at the temperature of maximal stability.

Considering these results together, it is reasonable to assume that the increase in LepFNR stability may be due to the existence of robust interactions between the rigid NADP^+^ binding subdomain and the FAD binding domain. To validate this hypothesis, we calculated the stability gained during the interaction between the FAD and NADP^+^ binding domains in different FNRs considering them as isolated molecules, preserving the original parameters of the PDB files. Using the PDBePISA tool (Protein Interfaces, Surfaces and Assemblies), available at European Bioinformatics Institutes, complexes between these domains were produced *in silico*. The parameters calculated for the productive complexes are shown in Table 3. LepFNR displayed the highest free energy of assembly dissociation and the highest solvation free energy gain due to the interaction assembly between both domains as compared with other FNRs. Moreover, LepFNR displayed the largest number of potential hydrogen bonds across the interface, which may also explain the observed stability in low pH (see Fig. 2, inset). As a control, the amino acids that were detected *in silico* to be involved in the interactions between the NADP^+^ and FAD binding domains of *L. interrogans* and *E. coli* FNRs were replaced by alanines. A ΔG^int^ of −18.2 kcal/mol was obtained for the LepFNR mutant, a value 9.4 kcal/mol higher than the one obtained for the wild-type structure (Table 3). Using FoldX and YASARA (www.yasara.org) a destabilization of 6.3 kcal/mol was detected for the mutant LepFNR with respect to the wild-type enzyme. In the case of *E. coli* FNR mutant, the analysis of the protein interface did not reveal any specific interactions that might result in the formation of stable structures between domains. These results indicate that the changes predicted considerably affect stability parameters. It has been proposed that the NADP^+^ domain dynamics in the maize leaf FNR increases in response to pH, particularly in the flexible subdomain, and that this change is related to an increase in the enzyme catalytic activity [39]. Our results are in agreement with these observations. LepFNR is more stable, displays a larger number of potential hydrogen bonds across the domain interface, as compared with the pea leaf FNR, and concomitantly increases the diaphorase activity as a function of pH with a lower slope than the pea leaf enzyme (Fig. 2A). The increased LepFNR pH stability may arise from an increase in electrostatic interactions between both FAD and NADP^+^ binding domains leading to stabilization of the native conformation and maintenance of structural cooperativity in FNR, as was previously suggested for the *T. gondii* enzyme [46].

**Table 3:** Parameters of interaction between FAD and NADP^+^ binding domains of the different FNRs^a^.

FNR	ΔG^int^ (kcal/mol)^b^	ΔG^diss^ (kcal/mol)^c^	N_HB_ d	N_SB_ e	Total interactions
*L. interrogans* (2rc5)	−27.6	12.4	29	8	37
Pea leaf (1qg0)	−19.1	8.2	22	2	24
Spinach leaf (1fnb)	−14.9	10.5	23	7	30
Maize root (1jb9)	−19.7	9.8	19	11	30
Maize leaf (1gaw)	−19.4	10.2	26	6	32
*E. coli* (1fdr)	−19.4	4.0	16	3	19
*P. falciparum* (2ok8)	−23.1	7.8	27	0	27
*Anabaena* (1que)	−17.0	4.8	18	5	23

a The parameters were calculated by using the PISA server tool as described in the Materials and Methods section.

b ΔG^int^ indicates the solvation free energy gained upon formation of the assembly and is calculated as the difference in the total solvation energy of the isolated and assembled structures. This value does not include the effect of satisfied hydrogen bonds and salt bridges across the interfaces of the assembly.

c ΔG^diss^ indicates the free energy of assembly dissociation, which corresponds to the free energy difference between dissociated and associated states. Positive ΔG^diss^ values indicate that an external driving force should be applied to dissociate the assembly.

d N_HB_ indicates the number of potential hydrogen bonds across the interface.

e N_SB_ indicates the number of potential salt bridges across the interface.

Therefore, the extent of interaction between both domains may be the main reason for the resistance to denaturing agents such as temperature, urea or guanidinium chloride as displayed by LepFNR. Aliverti et al. [45] designed two FNR chimeric flavoenzymes by swapping the structural domains of the spinach leaf FNR and the maize root FNR. These authors found that only one of the two possible combinations produced an active enzyme (leaf FAD binding domain and root NADP^+^ binding domain). This chimera was less stable than the parent enzymes due to a suboptimal fit between both domains. The authors have suggested that the contact area between domains of this chimera is expected to have a lower hydrophobic character than the original enzymes, making the domain-domain interaction less rigid.

It has been postulated that FNR may have evolved, as have other flavoenzymes, by the assembly of single domain proteins, which mainly are those that contain FAD and NADP^+^ binding sites [47]. An evolution of the interaction of these different domains should have occurred to fulfill the metabolic needs of the different organisms. FNR stability may have been improved by a selection that increased the inter-domain interactions, which can be more easily varied without affecting the cofactor and substrate binding. The generally accepted principle for protein structure is that stability-function tradeoffs exist and that proteins evolve primarily to optimize their function in vivo, with relatively little evolutionary pressure to increase stability unless this later property has some relevance [48], [49]. Considering that LepFNR was acquired by a lateral gene transfer event and that its primary structure is very different to those of plastidic FNRs, it is possible that evolution and selection pressures enhanced this particular stability. During their life cycle, pathogenic *Leptospira* survive in the environment, waiting for the reinfection of animal hosts [50], [51]. During the latent period of their life cycle, which is outside the host, pathogenic *Leptospira* neither multiply nor grow, and they suffer disruption of different structures as abundant blebbing and vesicle formation on membrane surfaces, which indicates their inability to synthesize proteins and/or repair membrane structures [52]. However, after infection of an appropriate host, *Leptospira* reproduces and disseminates by blood to many organs. Thus, *Leptospira* maintains all essential proteins that are relevant for host infection during the latent step of its life cycle. This may explain the acquisition of an enzyme with elevated stability.

Although the main role of LepFNR in *Leptospira* is still unknown, some possible mechanisms in which the enzyme may participate can be suggested. The ferredoxin/FNR redox system from *P. falciparum* serves as electron donor of LytB, that catalyzes the last step in the metabolism of 1-deoxy D-xylulose-5-phosphate [12]. This reaction participates in the alternative “non-mevalonate” pathway for the synthesis of isoprenoids [53]. This pathway might be one of the possible reasons for the existence of a highly efficient FNR in *Leptospira*, since it requires a significant contribution of reducing equivalents. Moreover, one of the enzymes of this pathway in *Thermosynechococcus elongatus*, the (E)-4-hydroxy-3-methylbutan-2-enyl diphosphate synthase, is ferredoxin-dependent and a NADPH reducing shuttle system with FNR can function as electron donor [54]. Genomic sequencing has showed the existence of the “non-mevalonate” pathway in *L. interrogans* [55]. Therefore, it can be suggested that LepFNR could provide electrons through the LB107 ferredoxin for the isoprenoids synthesis. This pathway has become an attractive target for the development of antibacterial compounds, especially for the fact that is present in clinically important pathogens and is not present in humans [15], [16], [56]. In vertebrates, a ferredoxin NADP(H) reductase (adrenodoxin NADPH reductase) transfers electrons from NADPH to specialized cytochromes P450 via a ferredoxin known as adrenodoxin. This pathway has been implicated in steroid hormone biosynthesis and in the cellular biogenesis of iron-sulfur clusters in mitochondria [15]. The adrenodoxin-NADPPH reductase belongs to the “two dinucleotide binding domains” flavoprotein superfamily [57] which includes the glutathione reductase and is completely unrelated to the plant-type FNR family to which LepFNR belongs. Important differences have been observed concerning the NADP(H) and the protein substrate binding modes between enzymes from these two families. These differences could be exploited for the development of drugs that differentially inhibit the plastidic-type FNR without affecting the mitochondrial enzyme. Several inhibitors have been described for FNR enzymes and the potential to development of new ones has been analyzed [15]. However, drugs that specifically inhibit plastidic FNRs have yet to be designed.

In the same way, one of the protection systems against bacterial infection in vertebrates consists in withholding of iron in plasma. To survive under these conditions bacteria have developed different mechanisms to obtain this metal as scavenging it from hemoglobin. *L. interrogans* is capable to grow using heme/hemoglobin as a sole iron source. The bacterium has a heme oxygenase that is expressed and relevant for the bacterial infection [25], [26]. The reaction catalyzed by the heme oxygenase is highly dependent on reducing power, since it requires NADPH and a hemoprotein reductase. This crucial function might be played by LepFNR in *Leptospira*, as it was already observed for the FNR enzyme of *Pseudomonas aeruginosa* [5].

Ferredoxins usually work as electron hubs providing reduction equivalents for multiple metabolic pathways. It is expected that the LepFNR/LB107 ferredoxin couple participates in a variety of metabolic reactions in *Leptospira*. Recently, it was shown that the very low potential [4Fe-4S] ferredoxin from *Pseudomonas aeruginosa* displays characteristics of a housekeeping protein. The same authors showed that deleting the gene that codes for this ferredoxin completely abolished bacterial growth, unless a plasmid copy of the gene was provided to the deleted strain [58].

Taking together the possible insertion of LepFNR in some of these relevant metabolic pathways in this pathogenic bacterium and the particular features concerning its high catalytic activity and significant structural stability make this enzyme a new potential antimicrobial target to investigate in *Leptospira*.

## Materials and Methods

### Data Base Search, Amino Acid Sequence Alignment and Bayesian phylogeny

The pea leaf FNR sequence from amino acids 53 to 360 (Genbank GI:119905) and the complete sequences of LepFNR (Genbank GI: 294828514), *E. coli* FNR (Genbank GI: 290446) and *Rhodobacter capsulatus* FNR (Genbank GI: 7025501) were used as query sequences to perform standard tBLASTn through the NCBI blast search engine of the complete DNA NCBI database (Release 181). The phylogenetic analysis was performed using 27 sequences and 303 representative amino acids as determined by the program Gblocks [59] after sequence alignment using T-Coffee [60]. The generated multiple alignments were used for obtaining a tree by the Bayesian inference method using the MrBayes package (v3.1.2) [61]. The fixed Jones model was used for amino acid substitution [62]. The Markov Chain Monte Carlo method was run with the following settings: 4 chains; 2,000,000 generations, sampling every 100 generations; and “burn-in” of 500 sampled trees discarded. Finally, a consensus tree was obtained (50% majority rule) and plotted using Dendroscope [63].

### Protein expression and purification

*LepFNR* was expressed and purified as described in [64]. Briefly, the vector pET32JO-LepFNR, which contained the cDNA sequence for the protein fused with the thioredoxin DNA, was expressed in *E. coli* BL21(DE3)pLysS and grown at 37°C in LB medium supplemented with the appropriated antibiotic. Expression was induced by the addition of 1 mM IPTG (final concentration) for 3 hours at 30°C. LepFNR was purified by Ni-NTA affinity chromatography and dialyzed against 50 mM Tris-HCl, pH 8.0, 150 mM NaCl. The fusion protein was digested with thrombin, and the thioredoxin was further removed by a subsequent Ni-NTA affinity chromatography procedure.

Ferredoxins LA4086 and LB107 from *L. interrogans* were overexpressed in *E. coli* C41 cells transformed with pET28Fd1 and pET28Fd2 vectors and the ISC operon expressing plasmid [32], as a fusion with a His6-tag containing a thrombin recognition site between the His6-tag and the first amino acid of each protein. The pET28Fd1 and pET28Fd2 expression vectors were constructed by inserting the coding sequence for *L. interrogans* LA4086 and LB107 into pET28-a. The coding sequences *L. interrogans* LA4086 and LB107 were amplified by PCR using the primers depicted in Supporting Table S1 and the genomic DNA from *L. interrogans* serovar Lai 56601, kindly provided by Dr Xiao-Kui Guo from the Dept. of Microbiology Shanghai Second Medical University, Shanghai, China, as a template. For functional expression bacteria were grown at 37°C in 1L LB medium supplemented with kanamycin (50 µg/ml) and tetracycline (15 µg/ml) for 3 h and then expression induced by the addition of 0.25 mM IPTG and supplemented with 2 mM ammonium ferric citrate. Then, the cultures were maintained during 16 h at 18°C with mild agitation. *E. coli* cells were collected by centrifugation, and disrupted by sonication in 50 mM Tris-HCl, pH 8.0, 75 mM NaCl, 0.5 mM DTT. Then, ferredoxin were purified by Ni-NTA affinity chromatography and dialyzed against the same buffer. For LB107 ferredoxin, all purification steps were carried out under a reduced oxygen atmosphere.

Pea leaf ferredoxin and *E. coli* ferredoxin and flavodoxins were purified as described [8], [65].

### Enzymatic assays

FNR-dependent diaphorase and cytochrome *c* reductase activities were determined using published methods [66]. Diaphorase activity was measured in 50 mM Tris–HCl, pH 8.0, which contained 1 mM potassium ferricyanide, 3 mM glucose-6-phosphate, 0.3 mM NADP^+^ and 1 unit of glucose-6-phosphate dehydrogenase. After the addition of 20 nM FNR, the reactions were spectrophotometrically monitored by following potassium ferricyanide reduction at 420 nm (ε_420_ = 1 mM^−1^ cm^−1^). The cytochrome *c* reductase activity of FNR, using either pea leaf ferredoxin, *E. coli* ferredoxin or flavodoxin A, was assayed in a reaction medium (1 ml) containing 50 mM Tris-HCl, pH 8.0, 0.3 mM NADP^+^, 3 mM glucose-6-phosphate, 1 unit of glucose-6-phosphate dehydrogenase and 50 µM cytochrome *c*. After the addition of 15–100 nM FNR, the reaction was spectrophotometrically monitored by following cytochrome *c* reduction at 550 nm (ε_550_ = 19 mM^−1^ cm^−1^). The cytochrome *c* reductase activity of FNR and *Leptospira* ferredoxins were performed essentially as describe above using 300 nM FNR and 50 mM Tris/Hepes as buffer for the reaction medium. The measurements were made under an argon atmosphere. All kinetic experiments were performed at 30°C.

Ferric reductase activity was performed in 0.2 ml of 50 mM Tris-HCl, pH 8.0, 0.3 mM NADPH, 0.3 mM ferric citrate, 1 mM ferrozine and 50 µM FNR. The reduction of ferrozine was followed through the decrease of the absorbance at 562 nm (ε_562_ = 28 mM^−1^ cm^−1^) under both aerobic and anaerobic conditions, the latter produced by bubbling argon in the reaction medium for 15 minutes.

Oxidase activity was performed by monitoring the consumption of NADPH at 340 nm (ε_340_ = 6.2 mM^−1^ cm^−1^) in 0.2 ml 50 mM Tris-HCl, pH 8.0, 0.3 mM NADPH and 50 µM FNR. The reaction was carried under both aerobic and anaerobic conditions.

### Determination of the dissociation constant of the LepFNR·NADP^+^ complex

Difference absorption spectroscopy was used to evaluate the dissociation constant of the LepFNR·NADP^+^ complex. The experiment was essentially performed as previously described [44] using 35 µM LepFNR in 50 mM Tris-HCl, pH 8.0, which was titrated with the substrate at 25°C.

### The effect of urea and guanidinium chloride on the catalytic activity

Enzyme samples were diluted at 6 µM in both the absence and the presence of increasing concentrations of urea and guanidinium chloride and incubated for 1 h at 25°C. Next, aliquots of these protein samples were diluted at 60 nM, and the diaphorase activity was measured at two different conditions. In the first assay, the medium contained 50 mM Tris-HCl, pH 8.0, 1 mM potassium ferricyanide, 0.3 mM NADPH and the same concentration of denaturing agent in which the enzyme was previously incubated. In parallel, the activity was measured in a similar medium that lacked the denaturing agent. The activity measured without pre-incubation with denaturing agent was considered 100% activity.

### Circular Dichroism Measurements

CD spectra were obtained using a JASCO J-810 spectropolarimeter at 25°C. The spectra were recorded in the far-UV region (200–250 nm) with 0.5 µM protein in a cell with 0.1 cm path length. The samples were previously filtered through G25 Sephadex spin columns equilibrated with 50 mM potassium phosphate, pH 8.0.

### Thermal unfolding transitions

Protein stock solutions were diluted to a final concentration of 0.5 µM in 50 mM potassium phosphate, pH 8.0. The CD signal was registered by excitation at 220 nm while the temperature of the sample was increased at a uniform rate of 1°C min^−1^ (from 15 to 80°C) on a Jasco J-810 spectropolarimeter equipped with a Peltier temperature controller system in a cell with 0.1 cm path length. Thermal unfolding transitions were analyzed assuming a two-state approximation in which only the native and unfolded states are significantly populated. The *T*_m_ and Δ*C*_p_ were determined by fitting the experimental data to the equations found below as described elsewhere [67]. The Solver complement of Microsoft Excel 2000 was used to fit the data to the equations developed by Primalov [68]:
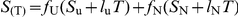


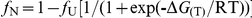


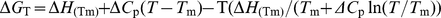

*S*_(T)_ is the value of the physical observable at temperature T; *f*_U_ and *f*_N_ are the unfolded and folded fractions, respectively; *S*_u_ and *S*_N_ are the extrapolated intrinsic signals of the unfolded and folded state at 0°K, respectively; *l*_u_ and *l*_N_ are the linear dependency of *S*_u_ and *S*_N_ on temperature, respectively; *T*_m_ is the midpoint transition temperature; Δ*H*_(Tm)_ is the enthalpy change of unfolding at temperature *T*_m_ and Δ*C*_p_, the heat capacity change [67].

### Fluorescence Spectroscopy

Fluorescence spectra were monitored using a Varian (Palo Alto, CA, USA) Cary Eclipse fluorescence spectrophotometer interfaced with a personal computer with in a cell with 1 cm path length. The measurements were carried out at 25°C with 3 µM protein in 50 mM Tris-HCl, pH 8.0. The FAD exposure was measured by fluorescence emission at 525 nm (λ excitation 450 nm) and increasing the urea concentration in 50 mM Tris-HCl, pH 8.0. Samples were previously filtered through G25 Sephadex spin columns equilibrated with 50 mM Tris-HCl, pH 8. The fluorescence displayed at 2% SDS (w/v) was considered as 100% FAD exposure, and the other fluorescence values registered at different urea concentrations were expressed as percentages of this value.

### Analysis of the interaction between the FAD and NADP^+^ binding domains

The PDB files of *L. interrogans* (2rc5), pea leaf (1qg0), spinach leaf (1fnb), maize root (1jb9), maize leaf (1gaw), *E. coli* (1fdr), *P. falciparum* (2ok8) and *Anabaena* (1que) FNRs were edited through the Swiss-Pdv Viewer 3.7 program to work with only one molecule. The N-terminal FAD binding domain of pea leaf FNR was considered from the first amino acid to methionine 151, and the NADP^+^ binding domain was defined from proline 152 to the C-terminal residue. FAD and NADP^+^ binding domains of all other enzymes were defined by superimposing its crystal structures to that of pea leaf FNR using amino acids 151 and 152 as the domain limits. Next, each domain was renamed as chain A and B by using the command “rename current layer” in the “edit” tool of Swiss-Pdv Viewer 3.7. The PDB files were saved and the parameters of domain-domain interaction were obtained by uploading these PDB files as coordinate files in PDBePISA (Protein Interfaces, Surfaces and Assemblies) [69] using the web server available (http://www.ebi.ac.uk/msd-srv/ssm/ssmstart.html).

## Supporting Information

Figure S1 **Multiple sequence alignment of the different FNRs.** Conserved sequences in plant-type ferredoxin-NADP(H) reductases that define this structural family are shaded in yellow (segments 1–6). The residues that interact with the adenosine moiety of FAD and are specific to the plastidic-type FNR are also shown in yellow and label as “A”. The common plastidic FNR motif SLCV(K/R)(R/Q)(L/A) [9] is shaded in grey, and the positively charged amino acids are in light blue. The letters in brackets indicate reductases from leaf (L), embryo (E) or root (R). The database accession numbers of different FNRs sequences are GB: NP_714507.2 for *Leptospira interrogans* serovar Lai str. 56601, GB: ABJ77386.1 for *Leptospira borgpetersenii* serovar Hardjo-bovis JB197, GB: YP_001840773.1 for *Leptospira biflexa* serovar Patoc strain ‘Patoc 1 (Paris)’, GB: YP_001632179.1 for *Bordetella petrii* DSM 12804, GB: ACD17089.1 for *Burkholderia phytofirmans* PsJN, GB: AAN39377.1 for *Azoarcus evansii*, GB: YP_004152444.1 for *Variovorax paradoxus* EPS, GB: XP_002371767.1 for *Toxoplasma gondii* ME49, SP: Q41014.2 for *Pisum sativum* (root isozyme), GB: BAC83340.1 for *Oryza sativa* (embryo isozyme), SP: O04397.1 for *Nicotiana tabacum* (root-type isozyme), GB: XP_002290014.1 for *Thalassiosira pseudonana* CCMP1335, GB: AAP79145.1 for *Bigelowiella natans*, GB: XP_001697352.1 for *Chlamydomonas reinhardtii* (leaf isozyme), GB: NP_925241.1 for *Gloeobacter violaceus* PCC 742, GB: EEE40085.1 for *Prochlorococcus marinus* str. MIT 9202, SP: P00455.1 for *Spinacia oleracea* (leaf isozyme), SP: P10933.1 for *Pisum sativum* (leaf isozyme, GB: ACG31602.1 for Zea mays (leaf isozyme), GB: CAA47015.1 for *Cyanophora paradoxa*, GB: YP_001518922.1 for *Acaryochloris marina* MBIC11017, GB: EAZ90412.1 for Cyanothece sp. CCY0110, GB: NP_441779.1 for *Synechocystis* sp. PCC 6803, GB: AAA91046.1 for *Anabaena variabilis*, GB: BAD97809.1 for *Nostoc commune*, GB: YP_171276.1 for *Synechococcus elongatus* PCC 6301, GB: XP_966214.1 for *Plasmodium falciparum* 3D7. GB: GenBank; SP: Swiss-Prot.
(PDF)

Figure S2 **Comparison of the crystal structures of maize leaf and** ***Leptospira*** **FNRs with a generated model of the BoxAB enzyme.** Superimposed view of the model for the BoxAB reductase carboxy terminal domain with the structures of (A) maize leaf FNR and (B) LepFNR. (C) Superimposed view of maize leaf FNR and LepFNR. The figure was drawn using Swiss-PdbViewer 3.7 and rendered with POV-Ray. Maize leaf FNR is represented in green, BoxAB in violet and LepFNR in red.
(TIF)

Figure S3 **Multiple sequence alignment of the root, leaf and** ***Leptospira*** **FNRs.** The database accession numbers of different FNRs sequences are those stated in Figure S1. The basic residues at positions 82 and 85, distinctive of leaf FNRs (numbers as in mature pea leaf FNR) are shaded in light blue. The letters in brackets indicate reductases from leaf (L) or root (R).
(PDF)

Figure S4 **Phylogenetic relationship between plastidic-type FNRs found in bacteria and photosynthetic organisms.** Multiple sequence alignment was obtained as described in the Materials and Methods section. Tree reconstruction was performed using the phylogenetic package PHYLIP 3.66 with the PROTDIST program selecting the Jones-Taylor-Thornton matrix option and the distance matrix NEIGHBOR program using a Neighbor-Joining clustering method. The robustness was tested by the bootstrap method using the SEQBOOT program and 5,000 sets of resampled sequences. The dashed line indicates an inference made in this work.
(TIF)

Figure S5 **Superimposed view of the LepFNR to the maize leaf FNR-ferredoxin complex.** View of the maize leaf bipartite FNR-ferredoxin complex (1gaq) with the ribbon diagram of ferredoxin colored in light blue, maize leaf FNR in green and LepFNR (2rc5) in pink. The box indicates the amino acid region in LepFNR that may interfere with the binding of pea ferredoxin. The figure was drawn using Swiss-PdbViewer 3.7 and rendered with POV-Ray structures.
(TIF)

Figure S6 **Sequence alignment for ferredoxins from different organisms.** (A) [2Fe-2S] Thioredoxin-like ferredoxins and (B) [4Fe-4S] ferredoxins [29]. (C) Alignment between the amino terminal ferredoxin component of BoxAB from *A. evansii* and the LB107 ferredoxin from *Leptospira interrogans*. Starred residues are completely conserved. Dots indicate decreasing the degree of conservation. The Fe-S cysteine ligands are shaded in green or violet.
(TIF)

Table S1 Nucleotide sequences of synthetic oligonucleotides used for the cloning and construction of the expression vectors of *Leptospira* ferredoxins.
(DOC)
