# Supplementary material for: A Highly Stable Plastidic-Type Ferredoxin-NADP(H) Reductase in the Pathogenic Bacterium Leptospira interrogans
Source: PLoS One. 2011 Oct 24;6(10):e26736. doi: 10.1371/journal.pone.0026736 (PMC3200346; doi:10.1371/journal.pone.0026736)
Supplement: Figure S1 — Multiple sequence alignment of the different FNRs. Conserved sequences in plant-type ferredoxin-NADP(H) reductases that define this structural family are shaded in yellow (segments 1–6). The residues that interact with the adenosine moiety of FAD and are specific to the plastidic-type FNR are also shown in yellow and label as “A”. The common plastidic FNR motif SLCV(K/R)(R/Q)(L/A) [9] is shaded in grey, and the positively charged amino acids are in light blue. The letters in brackets indicate reductases from leaf (L), embryo (E) or root (R). The database accession numbers of different FNRs sequences are GB: NP_714507.2 for Leptospira interrogans serovar Lai str. 56601, GB: ABJ77386.1 for Leptospira borgpetersenii serovar Hardjo-bovis JB197, GB: YP_001840773.1 for Leptospira biflexa serovar Patoc strain ‘Patoc 1 (Paris)’, GB: YP_001632179.1 for Bordetella petrii DSM 12804, GB: ACD17089.1 for Burkholderia phytofirmans PsJN, GB: AAN39377.1 for Azoarcus evansii, GB: YP_004152444.1 for Variovorax paradoxus EPS, GB: XP_002371767.1 for Toxoplasma gondii ME49, SP: Q41014.2 for Pisum sativum (root isozyme), GB: BAC83340.1 for Oryza sativa (embryo isozyme), SP: O04397.1 for Nicotiana tabacum (root-type isozyme), GB: XP_002290014.1 for Thalassiosira pseudonana CCMP1335, GB: AAP79145.1 for Bigelowiella natans, GB: XP_001697352.1 for Chlamydomonas reinhardtii (leaf isozyme), GB: NP_925241.1 for Gloeobacter violaceus PCC 742, GB: EEE40085.1 for Prochlorococcus marinus str. MIT 9202, SP: P00455.1 for Spinacia oleracea (leaf isozyme), SP: P10933.1 for Pisum sativum (leaf isozyme, GB: ACG31602.1 for Zea mays (leaf isozyme), GB: CAA47015.1 for Cyanophora paradoxa, GB: YP_001518922.1 for Acaryochloris marina MBIC11017, GB: EAZ90412.1 for Cyanothece sp. CCY0110, GB: NP_441779.1 for Synechocystis sp. PCC 6803, GB: AAA91046.1 for Anabaena variabilis, GB: BAD97809.1 for Nostoc commune, GB: YP_171276.1 for Synechococcus elongatus PCC 6301, GB: XP_966214.1 for Plasmodium falciparum 3D7. GB: [file pone.0026736.s001.pdf]

```
Leptospira interrogans       12 QINLFKKSNPYKAKVISNVLLTPETGTGKRPKKEGEALVHR--------IVLAIDHSA--YPYVIGQSGGVIPPGE-------DPE--KKAK-------- 84
Leptospira borgpetersenii    12 QINLFKKSNPYKAKVISNVLLTPEAGTGKRPKKEGEALVHR--------ITLALDHSA--YPYLIGQSGGVIPPGE-------DPE--KKAK-------- 84
Leptospira biflexa            5 QINLFKKSNPIQAQVLANTRLTPELGKGKRSMREGDAAVHR--------ITIAIDHSV--YPYMIGQSAGIIPPGL-------DPE--KQTK-------- 77
Bordetella petrii               YVNLYTHKTPALATVVGNYRVTDADTDS---------DIHH--------IVLEFGSVP--FPVLEGQSIGILPPGT-------DAQ--GRPH--------
Burkholderia phytofirmans       YVNLYTHKEPTSATVVGNYRLTDGSTDS---------DIHH--------IVLDFGSMP--FPVLEGQSIGILPPGT-------AAD--GRAH--------
Azoarcus evansii                YVNLYTPANPITATVTGNYRLTAEDASS---------DIHH--------IVLDFGTTP--FPVLEGQSIGIIPPGV-------DEK--GKPH--------
Variovorax paradoxus            YTNLFAPKSPTTATVVGNFNCTEAGFDS---------ETHH--------IVLDFGVVP--FPVLEGQSIGIVPPGV-------DAI--GKRH--------
Toxoplasma gondii            19 AVNTFRPASPLICRVVSVTPVTSKDSSP---------DDSHGEAPQVFSIVLHH-GKQ--LPFVEGQGIGIMPPSRAAQAPVADES--TSQR-------N 97
Pisum sativum(R)             24 PLNLHKPKEPYTATIVSVERLVGPKAPG---------ETCH--------IVIN-HDGN--VPYWEGQSYGVIPPGE-------NPKKPGSP--------- 87
Oryza sativa(E)              25 PLNLYKPKEPYTATIVSVERLVGPKAPG---------ETCH--------IVID-HGGN--VPYWEGQSYGVIPPGE-------NPKKPGSP--------- 88
Nicotiana tabacum(R)         24 PLNIYKPKEPYTATIVSVERLVGPKAPG---------ETCH--------IVID-HDGN--LPYWEGQSYGVIPPGE-------NPKKPGNP--------- 87
Thalassiosira pseudonana        PVNVYKNKAPFTGKVVSTKRIVGPQATG---------ETCH--------IVID-HQGN--FPYWEGQSWGVIPPGT-------REK-DGKP---------
Bigelowiella natans             PVNTHKNKAPLTAKVVSVERIVGPKATG---------ETCN--------IVID-HEGK--MPYWEGQSAGVIPPGI-------DPKR-NKP---------
Chlamydomona reinhardtii     62 PLNTYSNKAPFKAKVRSVEKITGPKATG---------ETCH--------IIIE-TEGK--IPFWEGQSYGVIPPGT-------KINSKGKE--------- 125
Gloeobacter violaceus        12 PVNIYRPASPFRGKALQNINLTPDDPDN---------DVRH--------VVLDLSGGD--LRYFEGQSIGIVPPGT-------DAQ--GKP--------- 74
Prochlorococcus marinus      79 PVNIYRPKTPYEGTVIENYSLLKEGAIG---------RVNH--------ITFDLKDSDPFLNYVEGQSIGIMPAGE-------DAN--GKP--------- 143
Spinacia oleracea (L)        28 TVNKFKPKTPYVGRCLLNTKITGDDAPG---------ETWH--------MVFS-HEGE--IPYREGQSVGVIPDGE-------DKN--GKP--------- 89
Pisum sativum(L)             22 VVNKFKPKEPYVGRCLLNTKITGDDAPG---------ETWH--------MVFS-TEGE--VPYREGQSIGIVPDGI-------DKN--GKP--------- 83
Zea mays (L)                 28 VTNLYKPKEPYVGRCLLNTKITGDDAPG---------ETWH--------MVFS-TEGK--IPYREGQSIGVIADGV-------DKN--GKP--------- 89
Cyanophora paradoxa          77 PLNLFRPANPYIGKCIYNERIVGEGAPG---------ETKH--------IIFT-HEGK--VPYLEGQSIGIIPPGT-------DKD--GKP--------- 138
Acaryochloris marina            PINIYRPNKPFVGKCISNEELVGPGGIG---------TCRH--------LIFDISAGD--LKYVEGQSIGIIADGT-------DDK--GKP---------
Cyanothece                      PVNIYKPKNPYIGKCVENYPLVAEGGSG---------IVQH--------LTFDISGGD--LHYLEGQSIGIIPPGT-------DDN--GKP---------
Synechocystis               126 PVNIYRPKTPYIGKVLENYPLVREGAIG---------TVQH--------LTFDLSAGD--LRYLEGQSIGIIPPGE-------DDK--GKP--------- 188
Anabaena variabilis          12 PVNLYRPNAPFIGKVISNEPLVKEGGIG---------IVQH--------IKFDLTGGN--LKYIEGQSIGIIPPGV-------DKN--GKP--------- 73
Nostoc commune                  PVNTYRPNAPFIGKVISNEPLVKEGGIG---------IVQH--------LKFDISAGD--LKYIEGQSIGIIPPGL-------DKN--GKP---------
Synechococcus elongatus     112 PVNIYRPNKPFVGKVLSNEPLVQEGGIG---------VVQH--------LTFDISEGD--LRYIEGQSIGIIPDGT-------DDK--GKP--------- 174
Plasmodium falciparum         6 FINLYTVKNPLKCKIVDKINLVRPNSPN---------EVYH--------LEIN-HNGL--FKYLEGHTCGIIPYYN-------ELD--NNPNNQINKDHN 76
```

```
                                                        1                          A                   2
Leptospira interrogans       85 ---------------GLADVGYTVRLYSIASPSYSFGMKEDNIEFIIKRDNIYDE---NGNIQFKGVCSNYMCDLKPGDEVTMTGPSGKKFLLPNT--DF 164
Leptospira borgpetersenii    85 ---------------GLADASYTVRLYSIASPSYSFGMKEDNIEFIIKRDNVYDE---NGNLQFKGVCSNYMCDLKPGDEVIMTGPSGKKFLLPAT--DF 164
Leptospira biflexa           78 ---------------GLADASYTVRLYSIASPSYSFGQTKDNIEFVVKRDNVYDE---NGNLLHKGVCSNYLCDLKPGDVVTMTGPAGKKFLLPQT--DF 157
Bordetella petrii               ----------------------HARQYSLASPRDGERAGYNNLSLTVKRVTED-----HQGQPARGVCSNYLCDLAKNDTVQVIGPFGNTFLMPN---HA
Burkholderia phytofirmans       ----------------------HARQYSIASPRDGERPGYNNVSLTVKRVSQQ------HGDSLDGVCSNYLCDLKKGDVVKVIGPFGGTFLMPN---HP
Azoarcus evansii                ----------------------LLRMYSVASPRDGERPHYNNLSLTVKRVVED-----HEGNPTRGVASNYVCDLKKGDKVQVTGPYGSTYLMPN---HP
Variovorax paradoxus            ----------------------HARQYSVASPRNGERPGYNNVSLTVKRVTED-----HQGDPVRGVCSNYVCDLKVGDTVQVVGPFGSSFLMPN---HP
Toxoplasma gondii            98 SVQPDGQGQQPTTVASICKRRLLPRIYSIASSRDGDDGCGSTLTLCVKKHIYADPVTGKRDRQKDGICSTYICDAKCGDEVEVTGPVGKALLLPT---ST 194
Pisum sativum(R)             88 ---------------------HNVRLYSIASTRYGDNFDGKTASLCVRRAVYYDPVTGKEDPSKNGVCSNFLCDSKPGDKIKIAGPSGKIMLLPED--DP 164
Oryza sativa(E)              89 ---------------------NTVRLYSIASTRYGDSFDGKTASLCVRRAVYYDPETGKEDPTKKGICSNFLCDSKPGDKVQITGPSGKIMLLPED--DP 165
Nicotiana tabacum(R)         88 ---------------------HNVRLYLIASTRYGDSFDGKTASLCVRRAVYYDPETGKEDPSKNGVCSNFLCDSKPGDKVKITGPSGKIMLLPEE--IP 164
Thalassiosira pseudonana        ---------------------HSVRLYSIASSRYGDDFTGNTGSLCVRRATYWCPELKADDPAKKGICSNFLCDTTAGDEVMMTGPAGKVMLMPEE--DP
Bigelowiella natans             ---------------------YGVRLYSIASTRYGDDFTGKTMTLCVRRATYWDPELGKEDPAKKGVCSNYLCDAKPGTELALTGPAGKVMLMPEE--KP
Chlamydomona reinhardtii    126 --------------------VPTARLYSIASSRYGDDGDGQTASLCVRRAVYVDPETGKEDPAKKGLCSNFLCDATPGTEISMTGPTGKVLLLPAD---A 202
Gloeobacter violaceus        75 ---------------------HKLRLYSIASSRIGDNKDGKTVSLCVKRVVYKHP---ETGEIVRGVASNFICDLAPGDDVSITGPTGKTFLLPED---P 147
Prochlorococcus marinus     144 ---------------------HKLRLYSIASTRHGDDYKGNTVSLCVRQLQYE-----KDGETINGVCSTYLCDIKPGDKVKITGPVGKEMLLPDE---E 214
Spinacia oleracea (L)        90 ---------------------HKLRLYSIASSALGDFGDAKSVSLCVKRLIYTN----DAGETIKGVCSNFLCDLKPGAEVKLTGPVGKEMLMPKD---P 161
Pisum sativum(L)             84 ---------------------HKLRLYSIASSAIGDFGDSKTVSLCVKRLVYTN----DAGEVVKGVCSNFLCDLKPGSEVKITGPVGKEMLMPKD---P 155
Zea mays (L)                 90 ---------------------HKVRLYSIASSAIGDFGDSKTVSLCVKRLIYTN----DAGEIVKGVCSNFLCDLQPGDNVQITGPVGKEMLMPKD---P 161
Cyanophora paradoxa         139 ---------------------HKLRLYSIASTRHGDFGDDKTVSLSVKRLEYTD----ANGNLVKGVCSNYLCDLKPGDEVMITGPVGTTMLMPED---Q 210
Acaryochloris marina            ---------------------HKIRLYSIASAHRGDYLDDKTVSLCVRQLEYPDP---DTGKTVYGVCSSFLCNLKPGDDVKITGPVGKEMLLPDD---P
Cyanothece                      ---------------------HKLRLYSIASTRHGDKLDDKTVSLCVRQLEYQHP---ETNETVYGVCSTHLCNLEVGADVAITGPVGKEMLLPDD---E
Synechocystis               189 ---------------------HKLRLYSIASTRHGDFGDDKTVSLCVRQLEYQN----EAGETVQGVCSTYLCNIKEGDDIAITGPVGKEMLLPPD---E 260
Anabaena variabilis          74 ---------------------EKLRLYSIASTRHGDDVDDKTISLCVRQLEYKHP---ESGETVYGVCSTYLTHIEPGSEVKITGPVGKEMLLPDD---P 147
Nostoc commune                  ---------------------EKLRLYSIASTRHGDDVDDKTVSLCVRQLEYKHP---ESGETVYGVCSTHLCFLEPGADVKITGPVGKEMLLPND---P
Synechococcus elongatus     175 ---------------------HKLRLYSIASTRHGDHVDDKTVSLCVRQLQYQN----EAGETINGVCSTFLCGLKPGDDVKITGPVGKEMLLPAD---T 246
Plasmodium falciparum        77 IINTTNHTNHNNIALSHIKKQRCARLYSISSSNN-----MENLSVAIKIHKYEQTEN-APNITNYGYCSGFIKNLKINDDIYLTGAHG-YFNLPNDAIQK 170
```

		3 … 4	
Leptospira interrogans	165	SGDIMFLAT**GTGIAP**FIGMSEELLEHKLI----------------------KFTGNITLVYGAPYSDELVMMDYLKGLESKH-KNF-KLITAI**SR**EEKNS	240
Leptospira borgpetersenii	165	EKDIMFLAT**GTGIAP**FIGMSEELLEHKLI----------------------KFTGNITLVYGAPYSDELVMMDYLRGLESKH-KNF-KLITAI**SR**EEKNP	240
Leptospira biflexa	158	SGDIFFFAT**GTGISP**FLGMVEELLVQKLI----------------------QFQGNLWLIYGAPYSDEIVLRDYFEDKTKEF-SNF-HFVTAI**SR**EEKNS	232
Bordetella petrii		RANLMMICT**GTGSAP**MRAMTERCRRRIER----------------------GTNGRLMLFFGARTQRELPYFGPLMKLPRDFI----DINLAL**SR**DASQP	
Burkholderia phytofirmans		NSHLLMICT**GTGSAP**MRAMTEYRRRRRLK----------------------GATGKLMLFFGARTKEELPYFGPLTNLPKDFI----DTNLAF**SR**TPGQP	
Azoarcus evansii		GSSIMMICT**GTGSAP**MRAMTERRRRRMDR----------------------KEGGELVLFFGARAPEELPYFGPLQKLPKEFI----DINFAF**SR**VPGEP	
Variovorax paradoxus		RSHIVMICT**GTGSAP**MRAMTEWRRRLR--------------------KSGKFEGGKLMLFFGARTQQELPYFGPLQSLPKDFI----DINLAF**SR**TPGQP	
Toxoplasma gondii	195	ETPLVMLAT**GTGVAP**FRSHLQALRRKLLSAGGP------------SAAQP-ANRPKVLLFIGARTAAAVPYMNEWRDIEAQRDGNFVDIHFAL**SR**QMKNP	281
Pisum sativum(R)	165	NATHIMIAT**GTGVAP**YRGYLRRMFMESV-------------------PTF-KFGGLAWLFLGVANVDSLLYDDEFTKYLKDYPDNF-RYNRAL**SR**EEKNK	243
Oryza sativa(E)	166	NATHIMIAT**GTGVAP**YRGYLRRMFMEDV-------------------PSF-KFGGLAWLFLGVANTDSLLYDEEFTNYLQQYPDNF-RYDKAL**SR**EQKNK	244
Nicotiana tabacum(R)	165	NATHIMIGT**GTGVAP**FRGYLRRMFMESV-------------------PT--KFNGLAWLFLGVANTDSLLYDDEFTKYLNDYPGNF-RYDRAL**SR**EQKNN	242
Thalassiosira pseudonana		KTDYIMVAT**GTGIAP**FRGFVRRLFFEST-------------------PAAKAYQGQAWLFLGVANSDALLYDDEFQEAKSKFPDNF-RLDYAL**SR**EQNNK	
Bigelowiella natans		ETPIIMLAT**GTGIAP**YRGFLRRLFVENT-------------------EAAEKFKGLAWLFLGVANSDALLYDEDWKAMQEKYPEKF-RYDVAL**SR**EQKNK	
Chlamydomona reinhardtii	203	NAPLICVAT**GTGIAP**FRSFWRRCFIENV-------------------PSY-KFTGLFWLFMGVGNSDAKLYDEELQAIAKAYPGQF-RLDYAL**SR**EQNNR	281
Gloeobacter violaceus	148	STNLVLIAT**GTGIAP**FRAFLRRIYDEM---------------------DT-PWQGKVWLFFGMQNSNSYLYQDELAGYTAK--GDF-EIVEAI**SR**EQKNA	222
Prochlorococcus marinus	215	DANIVMLAT**GTGIAP**MRAYLRRMFEATEKE----------------KNKW-NFKGKAWLFMGAPKSANLLYEEDLQRYLTDYPDNF-TYTKAI**SR**EQQNT	296
Spinacia oleracea (L)	162	NATIIMLGT**GTGIAP**FRSFLWKMFFEKH-------------------DDY-KFNGLAWLFLGVPTSSSLLYKEEFEKMKEKAPDNF-RLDFAV**SR**EQTNE	240
Pisum sativum(L)	156	NATVIMLGT**GTGIAP**FRSFLWKMFFEKH-------------------EDY-QFNGLAWLFLGVPTSSSLLYKEEFEKMKEKAPENF-RLDFAV**SR**EQVND	234
Zea mays (L)	162	NATIIMLAT**GTGIAP**FRSFLWKMFFEKH-------------------DDY-KFNGLGWLFLGVPTSSSLLYKEEFGKMKERAPENF-RVDYAV**SR**EQTNA	240
Cyanophora paradoxa	211	SATIIMLAT**GTGIAP**FRSFLRRMFEETH-------------------ADY-KFNGLAWLFLGVPTSSTLLYREELEKMQKANPNNF-RLDYAI**SR**EQTDS	289
Acaryochloris marina		NANIIMLGT**GTGIAP**FRSFLWHLFKENEDANANPFWLPKLLGWVKPSDKP-KFNGKTWLIFGVATTPNILYNKDLEDLQRRYPDNF-RLTKAI**SR**EQKNP	
Cyanothece		DANIVMLAT**GTGIAP**FRAFLWRMFFEKH-------------------EDY-KFKGKSWLIFGVPYTANILYKDKLEEIQTNYPDNF-ELTYAI**SR**EQKNS	
Synechocystis	261	DANIVMLAT**GTGIAP**FRAFLWRMFKEQH-------------------EDY-KFKGLAWLIFGIPKSENILYKDDLEKMAAEFPDNF-RLTYAI**SR**EQQNA	339
Anabaena variabilis	148	EANVIMLAT**GTGIAP**MRTYLWRMFKDAERA-AN--------------PEY-QFKGFSWLVFGVPTTPNILYKEELEEIQQKYPDNF-RLTYAI**SR**EQKNP	220
Nostoc commune		DANVIMLAT**GTGIAP**MRAYLWRQFKDAERA-AN--------------PEY-QFKGFSWLIFGVPTTPNLLYKEELEEIQQKYPDNF-RLTAAI**SR**EQKNP	
Synechococcus elongatus	247	DANVIMMGT**GTGIAP**FRAYLWRMFKDNERA-IN--------------SEY-QFNGKAWLIFGIPTTANILYKEELEALQAQYPDNF-RLTYAI**SR**EQKNE	329
Plasmodium falciparum	171	NTNFIFIAT**GTGISP**YISFLKKLFAYDKNNLYN--------------RNS-NYTGYITIYYGVYNEDSILYLNELEYFQKMYPNNI-NIHYVF**SY**KQ-NS	252

		5 … 6	
Leptospira interrogans	241	FDGGRMYISHRVREQAEAVKKILNGGGRF**YICGGP**KGMEKGVIEEIQKISGNTGT-YEEFKHHLEGAHQLFV**ETY**	314
Leptospira borgpetersenii	241	FDGGRMYISHRVREQAEIVKKILNGGGRF**YICGGP**KGMEKGVIEEIQKTAEHAGT-YEEFKHHLEGAHQLFV**ETY**	314
Leptospira biflexa	233	FDGGKMYITHRAKENAEAIKNAVNGNGKF**YICGGP**KGMEKGVIQEIMSACGTDST-YEAFKKHLEEKEQLFV**ETY**	307
Bordetella petrii		----RRYVQDLIRERAADVLDLLADPNTCI**YVCGL**KGMESGVLDALRDVVVQAGQDWTILHAALRREGRLHF**ETY**	
Burkholderia phytofirmans		----KRYVQDAMRERAVDVAHMLKDDNTHI**YVCGL**KGMEDGVLQALKEIGERHQLDWEALWVKLKKEGRLHL**ETY**	
Azoarcus evansii		----KRYVQDAIRERADKVFQMLQDDNCYI**YICGL**KGMEAGVLEAFRDICRAKGADWDALRPQLLSKARFHV**ETY**	
Variovorax paradoxus		----RRYVQDLMRERAADLAALLKDGASHF**YVCGL**KSMEEGVVLALRDVAKEAGLDWDTVGAALKREGRLHL**ETY**	
Toxoplasma gondii	282	-QGKKLYIQDVVWQEREKVWKALDRDGGHL**YACGL**KNMMVGVHEVLGNMAEEKGLPRDHLASLLKHQRRWHV**EVY**	355
Pisum sativum(R)	244	-NGGKMYVQDKIEEYSDEIFKLL-DNGAHI**YFCGL**RGMMPGIQETLKRVAEKRGESWEEKLSQLKKNKQWHV**EVY**	316
Oryza sativa(E)	245	-NGGKMYVQDKIEEYSDEIFKLL-DGGAHI**YFCGL**KGMMPGIQDTLKRVAEQRGESWEQKLSQLKKNKQWHV**EVY**	317
Nicotiana tabacum(R)	243	-KGGKMYVQDKIEEYSDEIFKLL-DEGAHI**YFCGL**KGMMPGIQDTLKRVAERRGESWEQKLSQLKKNKQWHV**EVY**	315
Thalassiosira pseudonana		-KGGKMYIQDKVEEYADEVFNKL-NNGAHI**YFCGL**KGMMPGIQDMLAEVCKSKGLDYDEWIKELKGKKQWHV**EVY**	
Bigelowiella natans		-SGGKMYIQDKVEEYGKEVFELM-NLGAHI**YFCGL**KGMMPGVLGAMESVAKSQGVNWEETLQKWKKAGQWHV**EVY**	
Chlamydomona reinhardtii	282	-KGGKMYIQDKVEEYADEIFDLL-DNGAHM**YFCGL**KGMMPGIQDMLERVAKEKGLNYEEWVEGLKHKNQWHV**EVY**	354
Gloeobacter violaceus	223	-QGGRMYVQHRIAEHAAALWELISGGNTYT**YICGL**KGMEDGIDDAFTAAAAAKDTVWKDYRYNLKQSGFWHV**ETY**	296
Prochlorococcus marinus	297	-KGGRMYIQDRVLESANELFNMIEDEKTHI**YLCGL**KGMEPGIDEAMTKAAEEKGLNWSELRPQLKKAGRWHV**ETY**	370
Spinacia oleracea (L)	241	-KGEKMYIQTRMAQYAVELWEMLKKDNTYF**YMCGL**KGMEKGIDDIMVSLAAAEGIDWIEYKRQLKKAEQWNV**EVY**	314
Pisum sativum(L)	235	-KGEKMYIQTRMAQYAEELWELLKKDNTFV**YMCGL**KGMEKGIDDIMVSLAAKDGIDWIEYKRTLKKAEQWNV**EVY**	308
Zea mays (L)	241	-AGERMYIQTRMAEYKEELWELLKKDNTYV**YMCGL**KGMEKGIDDIMVSLAEKDGIDWFDYKKQLKRGDQWNV**EVY**	314
Cyanophora paradoxa	290	-KGEKMYIQNRIAEYANEFWNMIQKPNTFV**YMCGL**RGMEDGIQQCMEDIAKANGTTWDAVVKGLKKEKRWHV**ETY**	363
Acaryochloris marina		-EGGRMYIQHRVAEHAEELWQMIQQENTHT**YICGL**KGMEKGIDEALSAVASKDGVNWSDYQREMKKAHRWHV**ETY**	
Cyanothece		-EGGRMYIQHRVAEQAEKLWNMLQDPKTHL**YMCGL**KGMEAGIEEGLKPFAEKQGVEWSDFVKGLKKEHRWHV**EVY**	
Synechocystis	340	-EGGRMYIQHRVAENAEELWNLMQNPKTHT**YMCGL**KGMEPGIDEAFTALAEQNGKEWTTFQREMKKEHRWHV**ETY**	413
Anabaena variabilis	221	-QGGRMYIQDRVAEHADELWQLIKNQKTHT**YICGL**RGMEEGIDAALSAAAAKEGVTWSDYQKDLKKAGRWHV**ETY**	304
Nostoc commune		-QGGRMYIQDRVAEHADELWQLIKNEKTHT**YICGL**RGMEEGIDAALTGAAAKEGVTWSDYQKELKKAHRWHV**ETY**	
Synechococcus elongatus	330	-AGGRMYIQDRVAEHADEIWNLLKDEKTHV**YICGL**RGMEDGIDQAMTVAAAKEDVVWSDYQRTLKKAGRWHV**ETY**	403
Plasmodium falciparum	253	-DATSFYVQDEIYKRKTEFLNLFNNYKCEL**YICG**HKSIRYKVMDILK----------SHDQFDEKKKKRVHV**EVY**	316
