# Supplementary figures and images for: A Highly Stable Plastidic-Type Ferredoxin-NADP(H) Reductase in the Pathogenic Bacterium Leptospira interrogans
Source: PLoS One. 2011 Oct 24;6(10):e26736. doi: 10.1371/journal.pone.0026736 (PMC3200346; doi:10.1371/journal.pone.0026736)

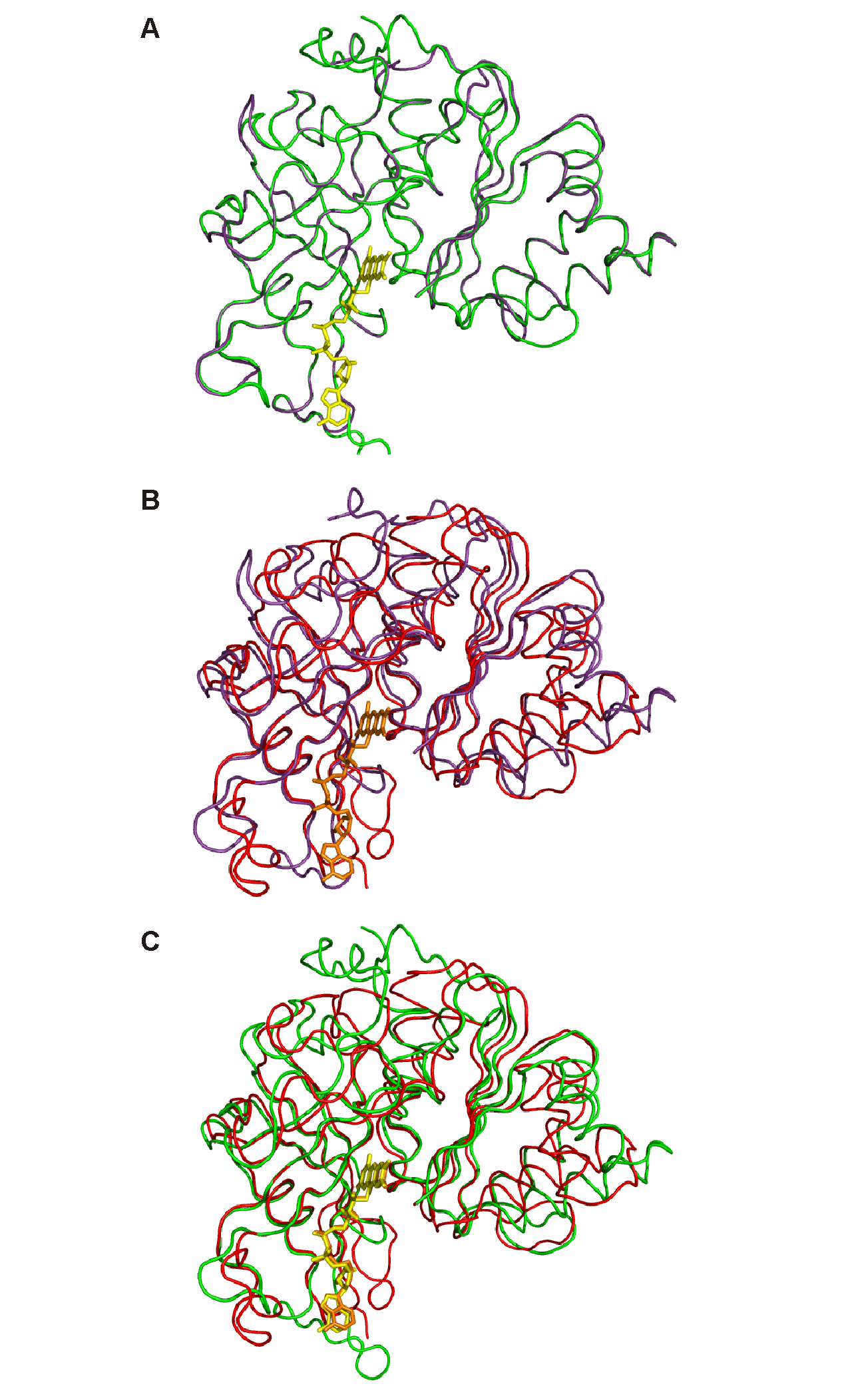

Supplement: Figure S2 — Comparison of the crystal structures of maize leaf and Leptospira FNRs with a generated model of the BoxAB enzyme. Superimposed view of the model for the BoxAB reductase carboxy terminal domain with the structures of (A) maize leaf FNR and (B) LepFNR. (C) Superimposed view of maize leaf FNR and LepFNR. The figure was drawn using Swiss-PdbViewer 3.7 and rendered with POV-Ray. Maize leaf FNR is represented in green, BoxAB in violet and LepFNR in red. (TIF) [file pone.0026736.s002.tif]

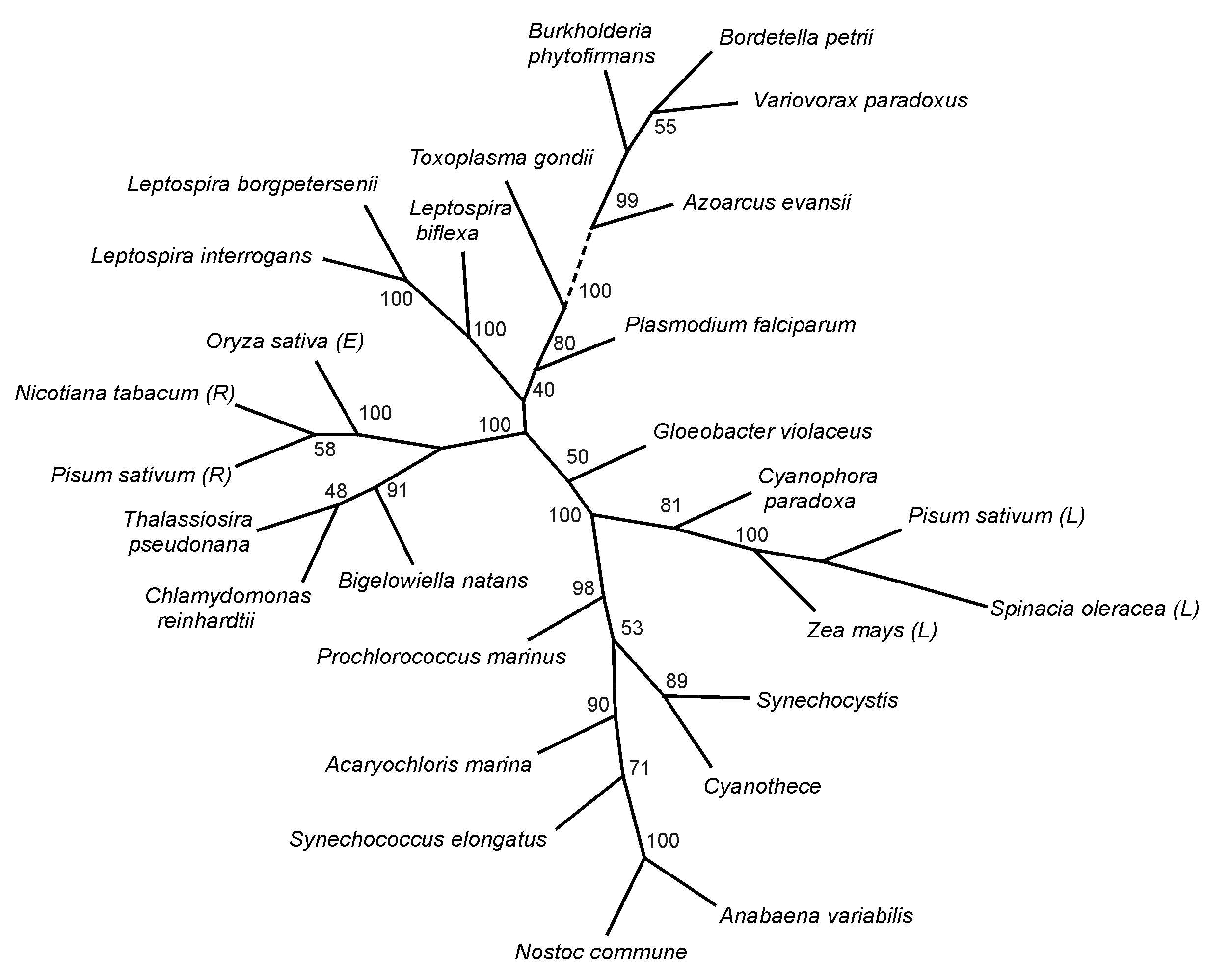

Supplement: Figure S4 — Phylogenetic relationship between plastidic-type FNRs found in bacteria and photosynthetic organisms. Multiple sequence alignment was obtained as described in the Materials and Methods section. Tree reconstruction was performed using the phylogenetic package PHYLIP 3.66 with the PROTDIST program selecting the Jones-Taylor-Thornton matrix option and the distance matrix NEIGHBOR program using a Neighbor-Joining clustering method. The robustness was tested by the bootstrap method using the SEQBOOT program and 5,000 sets of resampled sequences. The dashed line indicates an inference made in this work. (TIF) [file pone.0026736.s004.tif]

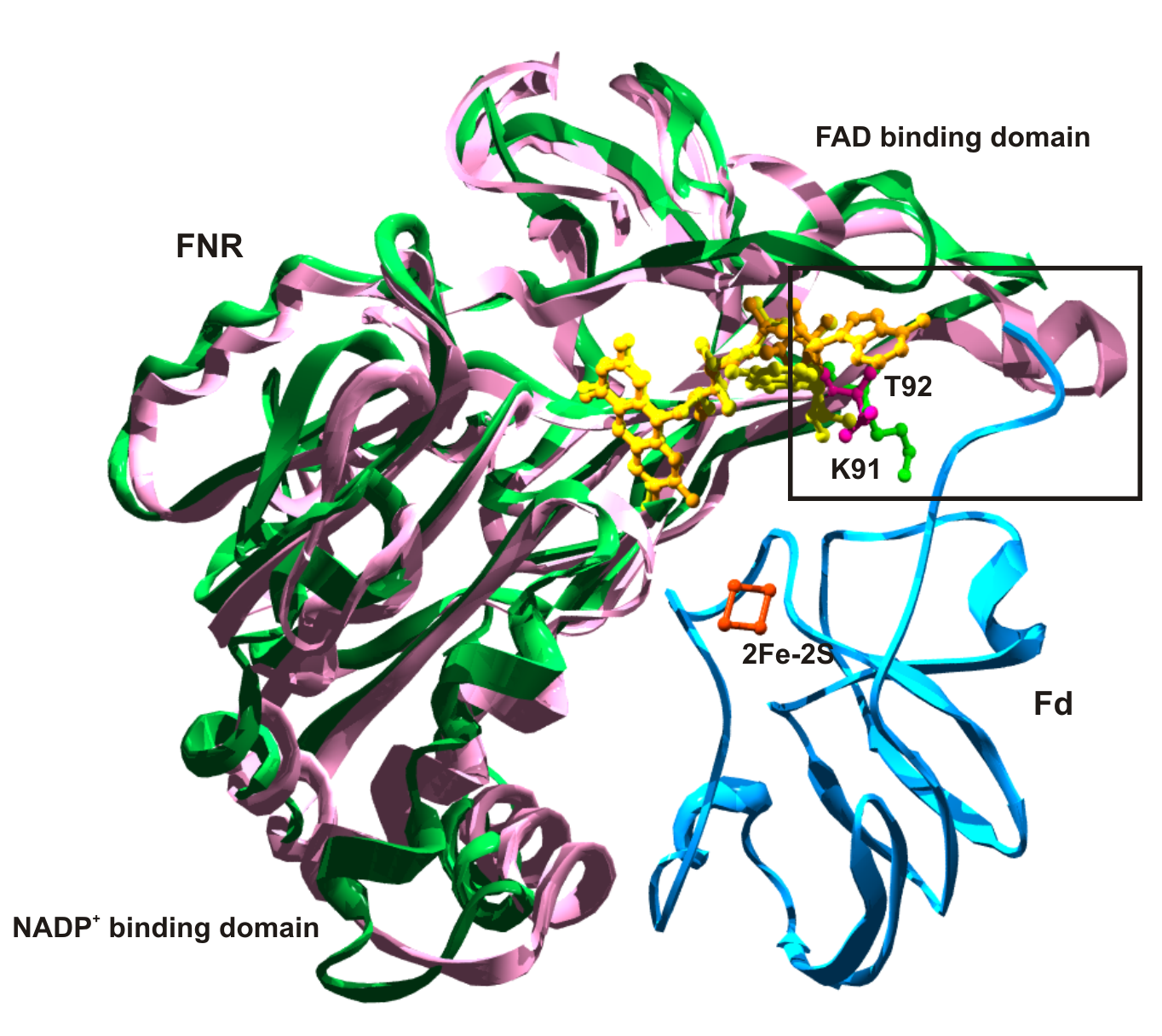

Supplement: Figure S5 — Superimposed view of the LepFNR to the maize leaf FNR-ferredoxin complex. View of the maize leaf bipartite FNR-ferredoxin complex (1gaq) with the ribbon diagram of ferredoxin colored in light blue, maize leaf FNR in green and LepFNR (2rc5) in pink. The box indicates the amino acid region in LepFNR that may interfere with the binding of pea ferredoxin. The figure was drawn using Swiss-PdbViewer 3.7 and rendered with POV-Ray structures. (TIF) [file pone.0026736.s005.tif]

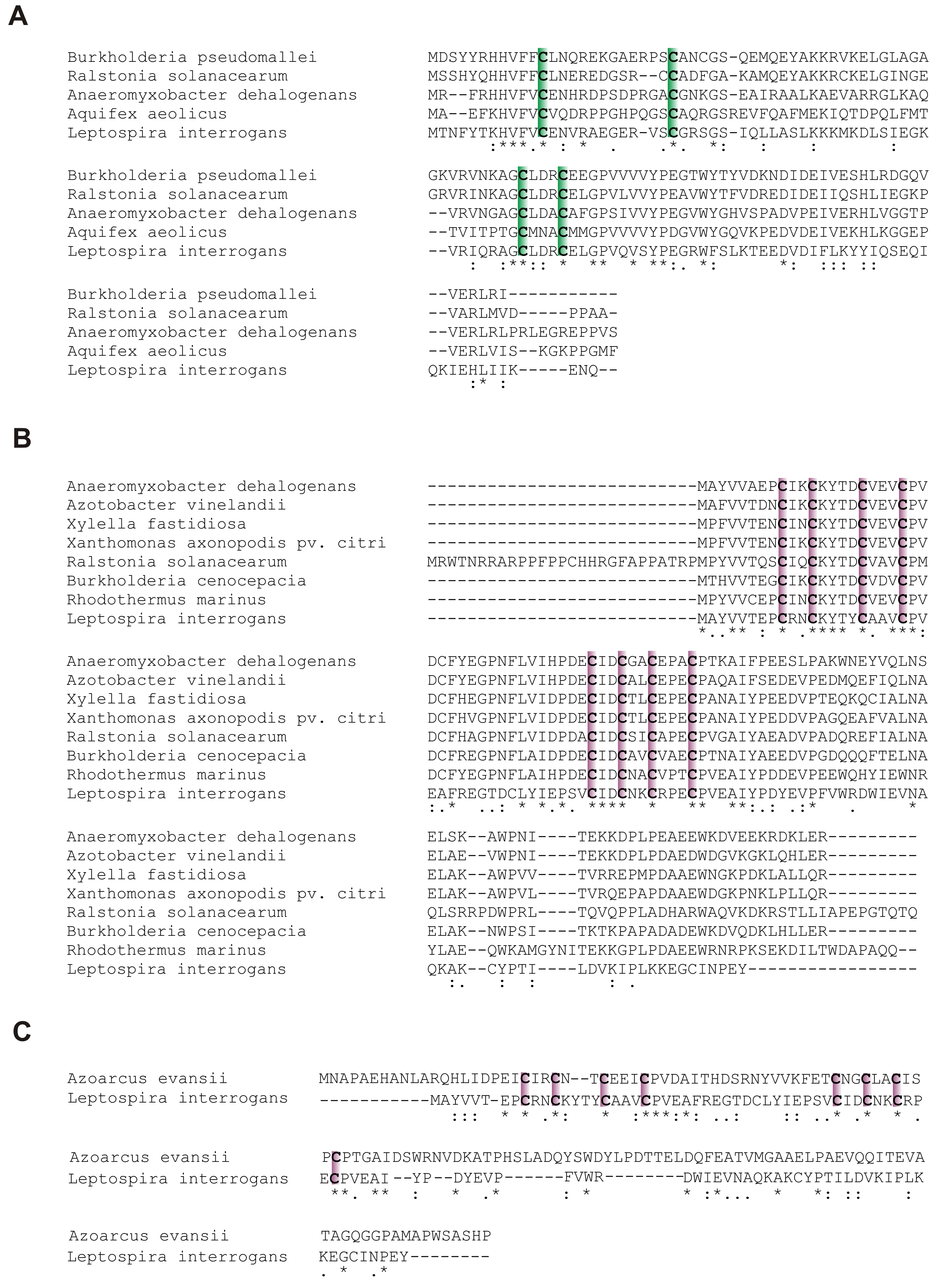

Supplement: Figure S6 — Sequence alignment for ferredoxins from different organisms. (A) [2Fe-2S] Thioredoxin-like ferredoxins and (B) [4Fe-4S] ferredoxins [29]. (C) Alignment between the amino terminal ferredoxin component of BoxAB from A. evansii and the LB107 ferredoxin from Leptospira interrogans. Starred residues are completely conserved. Dots indicate decreasing the degree of conservation. The Fe-S cysteine ligands are shaded in green or violet. (TIF) [file pone.0026736.s006.tif]
