# Supplementary material for: A Highly Stable Plastidic-Type Ferredoxin-NADP(H) Reductase in the Pathogenic Bacterium Leptospira interrogans
Source: PLoS One. 2011 Oct 24;6(10):e26736. doi: 10.1371/journal.pone.0026736 (PMC3200346; doi:10.1371/journal.pone.0026736)
Supplement: Figure S3 — Multiple sequence alignment of the root, leaf and Leptospira FNRs. The database accession numbers of different FNRs sequences are those stated in Figure S1. The basic residues at positions 82 and 85, distinctive of leaf FNRs (numbers as in mature pea leaf FNR) are shaded in light blue. The letters in brackets indicate reductases from leaf (L) or root (R). (PDF) [file pone.0026736.s003.pdf]

```
Spinacia oleracea (L)           28 TVNKFKPKTPYVGRCLLNTKITGDDAPG---------ETWHMVFSHEG-EIPYREGQSVGVIPDGED--KNGK-----PHKLRLYSIASSALGDFGDAKS 110
Pisum sativum (L)               22 VVNKFKPKEPYVGRCLLNTKITGDDAPG---------ETWHMVFSTEG-EVPYREGQSIGIVPDGID--KNGK-----PHKLRLYSIASSAIGDFGDSKT 104
Zea mays (L)                    28 VTNLYKPKEPYVGRCLLNTKITGDDAPG---------ETWHMVFSTEG-KIPYREGQSIGVIADGVD--KNGK-----PHKVRLYSIASSAIGDFGDSKT 110
Cyanophora paradoxa             77 PLNLFRPANPYIGKCIYNERIVGEGAPG---------ETKHIIFTHEG-KVPYLEGQSIGIIPPGTD--KDGK-----PHKLRLYSIASTRHGDFGDDKT 159
Anabaena variabilis             22 PVNLYRPNAPFIGKVISNEPLVKEGGIG---------IVQHIKFDLTGGNLKYIEGQSIGIIPPGVD--KNGK-----PEKLRLYSIASTRHGDDVDDKT 94
Pisum sativum (R)               24 PLNLHKPKEPYTATIVSVERLVGPKAPG---------ETCHIVINHDG-NVPYWEGQSYGVIPPGENPKKPGS-----PHNVRLYSIASTRYGDNFDGKT 108
Nicotiana tabacum (R)           24 PLNIYKPKEPYTATIVSVERLVGPKAPG---------ETCHIVIDHDG-NLPYWEGQSYGVIPPGENPKKPGN-----PHNVRLYLIASTRYGDSFDGKT 108
Oryza sativa (E)                25 PLNLYKPKEPYTATIVSVERLVGPKAPG---------ETCHIVIDHGG-NVPYWEGQSYGVIPPGENPKKPGS-----PNTVRLYSIASTRYGDSFDGKT 109
Leptospira interrogans          12 QINLFKKSNPYKAKVISNVLLTPETGTGKRPKKEGEALVHRIVLAIDHSAYPYVIGQSGGVIPPGEDPEKKAKGLADVGYTVRLYSIASPSYSFGMKEDN 111
Leptospira borgpetersenii       12 QINLFKKSNPYKAKVISNVLLTPEAGTGKRPKKEGEALVHRITLALDHSAYPYLIGQSGGVIPPGEDPEKKAKGLADASYTVRLYSIASPSYSFGMKEDN 111
Leptospira biflexa               5 QINLFKKSNPIQAQVLANTRLTPELGKGKRSMREGDAAVHRITIAIDHSVYPYMIGQSAGIIPPGLDPEKQTKGLADASYTVRLYSIASPSYSFGQTKDN 104
                                     * .:   *  .  :    :.   . *          . :: :        *  *** *::. * :  *       .:*** ***.  .     ..

Spinacia oleracea (L)          111 VSLCVKRLIYTN-DAGE---TIKGVCSNFLCDLKPGAEVKLTGPVGKEMLMPKD-PNATIIMLGTGTGIAPFRSFLWKMFFEKH----DDYKFNGLAWLF 201
Pisum sativum (L)              105 VSLCVKRLVYTN-DAGE---VVKGVCSNFLCDLKPGSEVKITGPVGKEMLMPKD-PNATVIMLGTGTGIAPFRSFLWKMFFEKH----EDYQFNGLAWLF 195
Zea mays (L)                   111 VSLCVKRLIYTN-DAGE---IVKGVCSNFLCDLQPGDNVQITGPVGKEMLMPKD-PNATIIMLATGTGIAPFRSFLWKMFFEKH----DDYKFNGLGWLF 201
Cyanophora paradoxa            160 VSLSVKRLEYTD-ANGN---LVKGVCSNYLCDLKPGDEVMITGPVGTTMLMPED-QSATIIMLATGTGIAPFRSFLRRMFEETH----ADYKFNGLAWLF 250
Anabaena variabilis             95 ISLCVRQLEYKHPESGE---TVYGVCSTYLTHIEPGSEVKITGPVGKEMLLPDD-PEANVIMLATGTGIAPMRTYLWRMFKDAERAANPEYQFKGFSWLV 191
Pisum sativum (R)              109 ASLCVRRAVYYDPVTGKEDPSKNGVCSNFLCDSKPGDKIKIAGPSGKIMLLPEDDPNATHIMIATGTGVAPYRGYLRRMFMESVP----TFKFGGLAWLF 204
Nicotiana tabacum (R)          109 ASLCVRRAVYYDPETGKEDPSKNGVCSNFLCDSKPGDKVKITGPSGKIMLLPEEIPNATHIMIGTGTGVAPFRGYLRRMFMESVP----T-KFNGLAWLF 203
Oryza sativa (E)               110 ASLCVRRAVYYDPETGKEDPTKKGICSNFLCDSKPGDKVQITGPSGKIMLLPEDDPNATHIMIATGTGVAPYRGYLRRMFMEDVP----SFKFGGLAWLF 205
Leptospira interrogans         112 IEFIIKRDNIYD-ENGN--IQFKGVCSNYMCDLKPGDEVTMTGPSGKKFLLPNTDFSGDIMFLATGTGIAPFIGMSEELLEHKLI------KFTGNITLV 202
Leptospira borgpetersenii      112 IEFIIKRDNVYD-ENGN--LQFKGVCSNYMCDLKPGDEVIMTGPSGKKFLLPATDFEKDIMFLATGTGIAPFIGMSEELLEHKLI------KFTGNITLV 202
Leptospira biflexa             105 IEFVVKRDNVYD-ENGN--LLHKGVCSNYLCDLKPGDVVTMTGPAGKKFLLPQTDFSGDIFFFATGTGISPFLGMVEELLVQKLI------QFQGNLWLI 195
                                    .: :::    .   *:      *:**.:: . :**  : ::** *. :*:*    .   :::.****::*      .::  .         :* *   *.

Spinacia oleracea (L)          202 LGVPTSSSLLYKEEFEKMKEKAPDNFRLDFAVSREQTNEK-GEKMYIQTRMAQYAVELWEMLKKDNTYFYMCGLKGMEKGIDDIMVSLAAAEGIDWIEYK 300
Pisum sativum (L)              196 LGVPTSSSLLYKEEFEKMKEKAPENFRLDFAVSREQVNDK-GEKMYIQTRMAQYAEELWELLKKDNTFVYMCGLKGMEKGIDDIMVSLAAKDGIDWIEYK 294
Zea mays (L)                   202 LGVPTSSSLLYKEEFGKMKERAPENFRVDYAVSREQTNAA-GERMYIQTRMAEYKEELWELLKKDNTYVYMCGLKGMEKGIDDIMVSLAEKDGIDWFDYK 300
Cyanophora paradoxa            251 LGVPTSSTLLYREELEKMQKANPNNFRLDYAISREQTDSK-GEKMYIQNRIAEYANEFWNMIQKPNTFVYMCGLRGMEDGIQQCMEDIAKANGTTWDAVV 349
Anabaena variabilis            192 FGVPTTPNILYKEELEEIQQKYPDNFRLTYAISREQKNPQ-GGRMYIQDRVAEHADELWQLIKNQKTHTYICGLRGMEEGIDAALSAAAAKEGVTWSDYQ 290
Pisum sativum (R)              205 LGVANVDSLLYDDEFTKYLKDYPDNFRYNRALSREEKNKN-GGKMYVQDKIEEYSDEIFKLLDNG-AHIYFCGLRGMMPGIQETLKRVAEKRGESWEEKL 302
Nicotiana tabacum (R)          204 LGVANTDSLLYDDEFTKYLNDYPGNFRYDRALSREQKNNK-GGKMYVQDKIEEYSDEIFKLLDEG-AHIYFCGLKGMMPGIQDTLKRVAERRGESWEQKL 301
Oryza sativa (E)               206 LGVANTDSLLYDEEFTNYLQQYPDNFRYDKALSREQKNKN-GGKMYVQDKIEEYSDEIFKLLDGG-AHIYFCGLKGMMPGIQDTLKRVAEQRGESWEQKL 303
Leptospira interrogans         203 YGAPYSDELVMMDYLKGLESKHKN-FKLITAISREEKNSFDGGRMYISHRVREQAEAVKKILNGGGRFYICGGPKGMEKGVIEEIQKISGNTG-TYEEFK 300
Leptospira borgpetersenii      203 YGAPYSDELVMMDYLRGLESKHKN-FKLITAISREEKNPFDGGRMYISHRVREQAEIVKKILNGGGRFYICGGPKGMEKGVIEEIQKTAEHAG-TYEEFK 300
Leptospira biflexa             196 YGAPYSDEIVLRDYFEDKTKEFSN-FHFVTAISREEKNSFDGGKMYITHRAKENAEAIKNAVNGNGKFYICGGPKGMEKGVIQEIMSACGTDS-TYEAFK 293
                                    *..    ::  : :    .     *:   *:***: :   * :**:  :  :    . :.    .    * :**  *:   :   .   .  :

Spinacia oleracea (L)          301 RQLKKAEQWNVEVY 314
Pisum sativum (L)              295 RTLKKAEQWNVEVY 308
Zea mays (L)                   301 KQLKRGDQWNVEVY 314
Cyanophora paradoxa            350 KGLKKEKRWHVETY 363
Anabaena variabilis            291 KDLKKAGRWHVETY 304
Pisum sativum (R)              303 SQLKKNKQWHVEVY 316
Nicotiana tabacum (R)          302 SQLKKNKQWHVEVY 315
Oryza sativa (E)               304 SQLKKNKQWHVEVY 317
Leptospira interrogans         301 HHLEGAHQLFVETY 314
Leptospira borgpetersenii      301 HHLEGAHQLFVETY 314
Leptospira biflexa             294 KHLEEKEQLFVETY 307
                                     *:   :  **.*
```
