# Supplementary material for: A Highly Stable Plastidic-Type Ferredoxin-NADP(H) Reductase in the Pathogenic Bacterium Leptospira interrogans
Source: PLoS One. 2011 Oct 24;6(10):e26736. doi: 10.1371/journal.pone.0026736 (PMC3200346; doi:10.1371/journal.pone.0026736)
Supplement: Table S1 — Nucleotide sequences of synthetic oligonucleotides used for the cloning and construction of the expression vectors of Leptospira ferredoxins. (DOC) [file pone.0026736.s007.doc]

**Table S1. Nucleotide sequences of synthetic oligonucleotides used for the cloning and construction of the expression vectors of *Leptospira* ferredoxins.**

| Amplified gene |  | Sequence |
| --- | --- | --- |
| [2Fe-2S] Ferredoxin (LA4086) |  | GGAATTC**CAT*ATG****ACGAACTTTTATACCAAACACG*  CGC**GGATCC**TAGAA*TCATTGGTTTTCCTTAATAATTAAATGTTC* |
| [4Fe-4S] Ferredoxin (LB107) |  | GGAATTC**CAT*ATG****GCTTATGTTGTTACAGAACC*  CGC**GGATCC**TCAGAA*CTAATATTCGGGATTTATACATC* |

*^a^* Italic letters indicate the complementary region to the genome template for hybridization during the PCR amplification. Bold letters indicate the recognition sites which were used for construction of the expression vectors.
